# Digital twin-enhanced three-organ microphysiological system for studying drug pharmacokinetics in pregnant women

**DOI:** 10.3389/fphar.2025.1528748

**Published:** 2025-02-12

**Authors:** Katja Graf, José Martin Murrieta-Coxca, Tobias Vogt, Sophie Besser, Daria Geilen, Tim Kaden, Anne-Katrin Bothe, Diana Maria Morales-Prieto, Behnam Amiri, Stephan Schaller, Ligaya Kaufmann, Martin Raasch, Ramy M. Ammar, Christian Maass

**Affiliations:** ^1^ Dynamic42 GmbH, Jena, Germany; ^2^ Placenta Lab, Department of Obstetrics, Jena University Hospital, Jena, Germany; ^3^ Institute of Biochemistry II, Center for Sepsis Control and Care, Jena University Hospital, Jena, Germany; ^4^ MPSlabs, ESQlabs GmbH, Saterland, Germany; ^5^ ESQlabs GmbH, Saterland, Germany; ^6^ Global Medical Affairs, Bayer Consumer Care AG, Basel, Switzerland; ^7^ Global R&D, Bayer Consumer Health, Steigerwald Arzneimittelwerk GmbH, Darmstadt, Germany; ^8^ Department of Pharmacology and Toxicology, Faculty of Pharmacy, Kafrelsheikh University, Kafr-El Sheikh, Egypt

**Keywords:** pregnancy, organ-on-chip, computational modelling, pharmacokinetics, safety

## Abstract

**Background:**

Pregnant women represent a vulnerable group in pharmaceutical research due to limited knowledge about drug metabolism and safety of commonly used corticosteroids like prednisone due to ethical and practical constraints. Current preclinical models, including animal studies, fail to accurately replicate human pregnancy conditions, resulting in gaps in drug safety and pharmacokinetics predictions. To address this issue, we used a three-organ microphysiological system (MPS) combined with a digital twin framework, to predict pharmacokinetics and fetal drug exposure.

**Methods:**

The here shown human MPS integrated gut, liver, and placenta models, interconnected via the corresponding vasculature. Using prednisone as a model compound, we simulate oral drug administration and track its metabolism and transplacental transfer. To translate the generated data from MPS to human physiology, computational modelling techniques were developed.

**Results:**

Our results demonstrate that the system maintains cellular integrity and accurately mimics *in vivo* drug dynamics, with predictions closely matching clinical data from pregnant women. Digital twinning closely aligned with the generated experimental data. Long-term exposure simulations confirmed the value of this integrated system for predicting the non-toxic metabolization of prednisone.

**Conclusion:**

This approach may provide a potential non-animal alternative that could contribute to our understanding of drug behavior during pregnancy and may support early-stage drug safety assessment for vulnerable populations.

## Highlights

• This study developed a three-organ model that simulates how drugs are absorbed, metabolized, and transferred across the gut, liver, and placenta, accurately representing drug behavior in pregnant women.
• Using this model with a digital twin simulation for prednisone, researchers can predict fetal drug exposure, helping ensure safety for vulnerable populations without animal testing.
• The results show that the system can provide early-stage insights into drug safety during pregnancy, paving the way for safer and more reliable drug assessments.

## 1 Introduction

Pregnant women represent a unique and vulnerable demographic in pharmaceutical research. The physiological changes during pregnancy, including altered metabolism, hormonal fluctuations, and the presence of the placenta, significantly impact the processes of drug absorption, distribution, metabolism, and excretion (ADME) (Hodge and Tracy, 2007), (Heikkinen et al., 2003). Despite these complexities, there is a critical lack of data regarding the safety and efficacy of medications in pregnant women, leaving both expectant mothers and their healthcare providers in a precarious position when it comes to drug therapy. Pregnant women are widely excluded from clinical trials due to the high risks posed to unborn children, including concerns about teratogenicity, unknown long-term effects on fetal development, and potential complications to maternal health (Sewell et al., 2022). Additionally, stringent regulatory guidelines, ethical principles protecting vulnerable populations, and liability concerns further limit their participation (Kaye, 2019). This exclusion creates significant knowledge gaps regarding how drugs behave in pregnancy, potentially leading to adverse events that were not predicted during preclinical development (Blehar et al., 2013), (Chaphekar et al., 2021). Traditional models, including animal studies and static cell cultures, fail to accurately capture the intricate physiological conditions of human pregnancy, leading to suboptimal predictions of maternal-fetal drug kinetics and safety (Wang et al., 2021). Advanced *in vitro* platforms such as organ-on-chip (OOC) represent a new hope for medically underrepresented groups. They can replicate mechanical forces and tissue-specific structures and activate important cellular mechanisms that are often neglected in conventional *in vitro* approaches and represent an opportunity for increased drug safety in these groups.

A key example of this challenge is prednisone (Xia et al., 2024), a synthetic corticosteroid commonly prescribed for its anti-inflammatory and immunosuppressive properties. Although it is classified as an FDA category C drug, meaning animal studies have shown adverse effects on the fetus while human data remain inconclusive, it is still prescribed when the benefits are believed to outweigh potential risks. Clinical use of prednisone during pregnancy has been associated with risks such as pre-term delivery and impaired fetal growth, potentially linked to drug kinetics across the maternal-fetal interface (Ponticelli and Moroni, 2018). Prednisone’s pharmacokinetics and pharmacodynamics (PK/PD) are complicated by its metabolism into the active metabolite prednisolone in the maternal liver. Prednisolone can cross the placenta, but its transfer is limited by the placental barrier and further enzymatic degradation on the fetal side, which helps to reduce fetal exposure (Beitins et al., 1972), (Turkay et al., 2012), (Levitz et al., 1978), (Blanford and Murphy, 1977). Understanding these dynamics, including how much prednisolone crosses into the fetal circulation and whether enzymatic mechanisms provide adequate protection, is critical for assessing the drug’s safety during pregnancy.

To address these complexities and bridge the gaps in our understanding of prednisone’s behavior during pregnancy, recent advances in organ-on-chip and microphysiological systems (MPS) offer promising alternatives to traditional models (Shroff et al., 2022). These *in vitro* platforms simulate the dynamic environment of human organs on a microscale, using human cells to mimic key physiological processes in a controlled setting. One of the most promising applications of MPS technology is in drug safety testing for underrepresented populations, such as pregnant women. Placenta-on-chip models, for example, allow researchers to replicate the maternal-fetal interface and study the transfer of substances, such as prednisolone, across the placental barrier.

Building on this technology, our study focuses on a three-organ model integrating gut- (GOC) (Kaden et al., 2024), (Maurer et al., 2019), liver- (LOC) (Kaden et al., 2023a), (Gröger et al., 2016), (Siwczak et al., 2022), and placenta-on-chip (POC) (Pemathilaka et al., 2022), (Kreuder et al., 2020), (Luconi et al., 2022), (Mosavati et al., 2020). The POC replicates the maternal-fetal interface, which enables a more comprehensive analysis of drug pharmacokinetics in pregnancy. This system allows us to simulate the oral administration of prednisone, its absorption through the gut, hepatic metabolism into prednisolone, and the transfer of both compounds across the placenta. The gut-liver connection simulates first-pass metabolism, which is crucial for understanding how drugs such as prednisone are processed before they reach the systemic circulation (Tsamandouras et al., 2017a), (Docci et al., 2022). The POC component, composed of trophoblast and fetal endothelial cells, enables us to study the maternal-fetal transfer of prednisone and prednisolone and assesses the protective role of the placental barrier and fetal enzymes in mitigating fetal drug exposure.

Although these MPS provide a more accurate simulation of pregnancy conditions compared to traditional models, it remains unclear how *in vitro* findings may be used to predict fetal drug exposure. While a few exemplary studies exist (Tsamandouras et al., 2017a), (Maass et al., 2019), (Ingber, 2022), (McAleer et al., 2019), a context-of-use-specific, integrated, and translational approach is still lacking, hindering the widespread adoption of MPS in drug development. Computational modelling techniques such as physiologically-based pharmacokinetic (PBPK) models (Dallmann et al., 2018a), (Lippert et al., 2019) may provide useful approaches to enabling MPS-to-human translation, providing guidance for aspects of dosing regimen, and predicting toxicity profiles.

In this study, we describe the development and implementation of a three-organ (gut-liver-placenta) MPS, combined with PBPK modeling, to evaluate the pharmacokinetics of prednisone and its active main metabolite, prednisolone, during pregnancy. Our results show that while prednisone crosses the placental barrier, transfer of prednisolone is limited by the blood-placenta barrier on the fetal side. These protective mechanisms reduce fetal exposure to potentially non-toxic levels of prednisolone. Additionally, our model demonstrates that simulated prednisone levels within the fetus are well below established toxicity thresholds. This supports the notion that this system can serve as an early-stage decision-making tool in drug development, offering a safer, faster, and more reliable alternative to traditional animal models.

## 2 Materials and methods

### 2.1 Ethics statement

Human peripheral blood was collected from healthy volunteers after receiving written, informed consent. This study was performed following the principles outlined in the Declaration of Helsinki. The blood donation protocol and use of blood for this study were approved by the institutional ethics committee of the Jena University Hospital (permission number 2207–01/08).

### 2.2 Cell culture

Cell culture and incubation procedures were performed at 37°C and 5% CO_2_ in a cell culture incubator, unless stated otherwise. They were sub-cultured (when reached 80% of confluence) using trypsin-EDTA solution (Capricorn Scientific, Ebsdorfergrund, Germany). Cells used in this study were tested negative for *mycoplasma* using the Venor®GeM Classic kit (Minerva Biolabs, Berlin, Germany).

Primary Human Umbilical Vein Endothelial Cells were obtained from Promocell (single donor, C-12200) seeded at a density of 2 × 10^4^ cells/cm^2^ in ECGM MV (Promocell, Heidelberg, Germany) with supplements and 1% penicillin/streptomycin (Gibco/Thermo Fisher Scientific). Medium exchange was performed every 3 days until seeding into biochips.

Caco-2 cells (acCELLerate GmbH, Hamburg, Germany) were seeded at a density of 2 × 10^4^ cells/cm^2^ and were cultured in DMEM with 4.5 g/L glucose (Lonza, Cologne, Germany) supplemented with 10% fetal calf serum (FCS, Capricorn Scientific), 1X MEM non-essential amino acids (Capricorn Scientific), 1 mM sodium pyruvate solution (Capricorn Scientific), 5 mg/mL holo-transferrin (Merck, Darmstadt, Germany) and 1X AAS. Medium was exchanged every three to 4 days until further seeding into biochips.

Human peripheral blood was collected in EDTA K3E tubes (Sarstedt, Nürmbrecht, Germany) and Serum Gel CAT tubes (Sarstedt) from healthy volunteers. Serum was obtained from each donor by centrifugation at 2,000 g for 10 min at room temperature (RT) and was stored at −20°C until use as supplement in cell culture medium.

Peripheral blood mononuclear cells (PBMCs) were isolated from EDTA blood by density gradient centrifugation as previously described (Kaden et al., 2023a). using lymphocyte separation medium (Capricorn Scientific). PBMCs were seeded into 6-well plates at a density of 1 × 10^6^ cells/cm^2^ in X-VIVO 15 medium (Lonza) supplemented with 10% human autologous serum and 1X AAS. After 1 h of incubation, PBMCs were washed twice with X-VIVO 15 medium without supplements to remove non-adherent cells. Subsequently, adherent monocytes were differentiated to monocyte-derived macrophages (MDMs) in X-VIVO 15 medium supplemented with 10% human autologous serum, 10 ng/mL macrophage colony-stimulating factor (M-CSF, Peprotech, Hamburg, Germany), 10 ng/mL granulocyte-macrophage colony-stimulating factor (GM-CSF, Peprotech) and 1X AAS for 5 days before seeding into biochips.

Undifferentiated HepaRG cells were obtained from Biopredic International (Rennes, France) and were cultured as described before (Gripon et al., 2002). Cells were seeded at a density of 2.7 × 10^4^ cells/cm^2^ in culture flasks and were cultured in William’s Medium E (Gibco/Thermo Fisher Scientific, Darmstadt, Germany) containing 10% FCS, 2 mM L-glutamine (Gibco/Thermo Fisher Scientific), 5 μg/mL insulin (Merck), 50 µM hydrocortisone-hemisuccinate (Biomol, Hamburg, Germany) and 1X AAS. The cells were cultured for 2 weeks with medium exchange every three to 4 days before HepaRG differentiation was initiated. Differentiation was induced by adding 2% dimethyl sulfoxide (DMSO, Merck) to the culture medium for at least 2 weeks as previously described (Gripon et al., 2002), (Cerec et al., 2007). Only fully differentiated HepaRG cells between passage 15-20 were used for biochip experiments.

Expandable human upcyte liver sinusoidal endothelial cells (LSECs) were purchased from Biotrend Chemikalien GmbH (Cologne, Germany). The cells were freshly thawed and seeded at a density of 1.3 × 10^4^ cells/cm^2^ on collagen A (PAN-Biotech, Aidenbach, Germany)-coated culture flasks. LSECs were cultured in supplemented ECGM MV with 1X AAS as previously described (Kaden et al., 2023b). Medium exchange was performed every two to 3 days until seeding into biochips. LSECs were used up to passage 2.

The first-trimester (choriocarcinoma) trophoblastic cell line BeWo was purchased from the American Type Culture Collection (ATCC, Manassas, VA, USA) and was cultured in F-12K medium (Gibco/Thermo Fisher Scientific) containing 10% FCS, and 1% penicillin/streptomycin (Gibco/Thermo Fisher Scientific) and seeded at a density of 4 × 10^4^ cells/cm^2^. Medium was exchanged every 3 days and prior to chip experiments, BeWo cells were gradually conditioned to the ECGM MV medium in proportions of 50%/50%, 75%/25%, and 100%/0% (ECGM MV/F-12K) every 24 h.

### 2.3 Biochip fabrication

Biochips (BC001 for the placenta model, BC002 for gut and liver models) were manufactured by Dynamic42 GmbH (Jena, Germany) from injection-molded polybutylene terephthalate (PBT) bodies. Biochips contain two individual culture chambers used for experimental duplicates. Each culture chamber is composed of a top and a bottom cell culture channel with adjacent microfluidic channels occupying an area of 2.18 cm^2^ top and 1.62 cm^2^ bottom for BC002 and, 1.80 cm^2^ top and 1.11 cm^2^ bottom for BC001, respectively. Both channels are separated by a 12 µm polyethylene terephthalate (PET) membrane with a pore density of 1 × 10^5^ pores/cm^2^ and a pore diameter of 8 µm (TRAKETCH Sabeu, Radeberg, Germany) for BC002 and a pore density of 0.6 × 10^6^ pores/cm^2^ and a pore diameter of 3 µm for BC001, respectively. The total volume with the corresponding ports was 290 µL for the top channel and 270 µL for the bottom channel for BC002 and, 350 µL for the top channel and 300 µL for the bottom channel for BC001, respectively. For cell seeding, 200 µL of cell suspension was introduced in the top channel and 150 µL in the bottom channel for BC002 and, 220 µL in the top channel and 200 µL in the bottom channel for BC001, respectively.

### 2.4 Gut model assembly

Gut models were assembled as previously described (Maurer et al., 2019). Prior to cell seeding, biochips were sterilized with 70% ethanol (Nordbrand Nordhausen GmbH, Nordhausen, Germany) and washed twice with AQUA AD injectabilia (B.Braun, Melsungen, Germany) and once with phosphate buffered saline (PBS, Capricorn). HUVECs were seeded in top channels of BC002 coated with 50 μg/mL collagen A at a density of 1.85 × 10^5^ cells/cm^2^ in ECGM MV with supplement and 1X AAS to form a confluent endothelial cell layer. Biochips were incubated for 24 h to facilitate attachment to the membrane. HUVECs were cultured statically for 48 h with daily medium exchange. For subsequent co-culture of HUVECs and MDMs, vascular perfusion medium (VPM) was prepared by addition of 10% autologous donor serum, 10 ng/mL M-CSF and 10 ng/mL GM-CSF to the supplemented ECGM MV with 1X AAS. MDMs were seeded at a density of 6.17 × 10^4^ cells/cm^2^ (1 × 10^5^ cells in total) in VPM on top of the HUVECs to complete the vascular cell layer. Vascular cell layers were incubated statically for further 24 h. Subsequently, Caco-2 cells were seeded in the opposing bottom channel coated with 50 μg/mL collagen A at a density of 7.63 × 10^4^ cells/cm^2^ in gut seeding medium (GSM) containing DMEM with 4.5 g/L glucose supplemented with 20% FCS, 1X MEM non-essential amino acids, 1 mM sodium pyruvate solution, 5 mg/mL holo-transferrin and 1X AAS. Biochips were incubated upside down for 24 h before perfusion was initiated. The medium in the Caco-2 channel was changed to gut perfusion medium (GPM) consisting of GSM with only 10% FCS 24 h post seeding.

### 2.5 Liver model assembly

Sterilized and collagen A-coated (50 μg/mL) BC002 biochip channels were gradually seeded with LSECs, MDMs, and differentiated HepaRG cells as previously described (Kaden et al., 2023a). The following cell densities were selected based on cell number ratios found in the liver. Concisely, human LSECs were seeded in top channels at a density of 0.45 × 10^5^ cells/cm^2^ and were cultured statically in supplemented ECGM MV with 1X AAS until reaching confluency. Medium was exchanged to vascular perfusion medium (VPM, see gut model) prior to the seeding of MDMs. Human MDMs were seeded on top of the LSECs at a density of 0.23 × 10^5^ cells/cm^2^ in VPM. MDMs and LSECs were cultured statically for further 24 h. Following this, differentiated HepaRG cells were seeded in the opposite hepatic channel at a density of 1.85 × 10^5^ cells/cm^2^ in hepatic thawing and seeding medium (HTSM) containing William’s Medium E, 5% FCS, 4% hepatocyte thawing and plating supplements CM3000 (Gibco/Thermo Fisher Scientific), 1 µM dexamethasone (Gibco/Thermo Fisher Scientific), 2 mM L-glutamine, 5 μg/mL insulin, 5 µM hydrocortisone-hemisuccinate (Merck) and 1X AAS. Adhesion of the cells to the membrane was facilitated by upside down incubation of the biochip for 24 h. HTSM was replaced by hepatic perfusion medium (HPM) composed of William’s Medium E, 10% FCS, 3.6% hepatocyte maintenance supplements CM4000 (Gibco/Thermo Fisher Scientific), 0.1 µM dexamethasone, 2 mM L-glutamine, 5 μg/mL insulin, 5 µM hydrocortisone-hemisuccinate, 0.1% DMSO and 1X AAS after 24 h. The model was cultured statically for further 24 h with medium exchange in both channels before applying vascular perfusion.

### 2.6 Placenta model assembly

BC001 biochip chambers were also sterilized with 70% ethanol and washed twice with ultra-pure distilled water (Invitrogen/Thermo Fisher Scientific) and gradually seeded with BeWos and HUVECs as reported previously (Blundell et al., 2016). HUVECs were seeded at a density of 0.45 × 10^5^ cells/cm^2^ in the bottom channel in ECGM MV with 1% penicillin/streptomycin (Gibco/Thermo Fisher Scientific) to recapitulate the fetal vasculature, and the biochip was incubated upside down for 5 h to facilitate cell attachment to the membrane. BeWo cells were seeded at a density of 0.23 × 10^5^ cells/cm^2^ in ECGM MV with 1% penicillin/streptomycin into the top channel to mimic the trophoblast layer at the maternal side. The biochip was maintained under static culture conditions for 24 h and the medium was exchanged before connection to perfusion.

### 2.7 Perfusion setup

For perfusion of the biochips, microfluidic medium reservoirs (Mobicol, Göttingen, Germany and microfluidic ChipShop, Jena, Germany) were attached to the channel ports (for hepatic models to the upper channel with LSECs and MDMs, for gut models to the upper channel with HUVECS and MDMs and the bottom Caco-2 channel, and for the placenta models to the upper BeWo channel). Biochips were connected to REGLO ICC peristaltic pumps (Masterflex/Ismatec, VWR International, Bruchsal, Germany) by silicon tubing (Dynamic42 GmbH, Jena, Germany).

In gut models, top channels with HUVECs and MDMs were perfused with VPM and Caco-2 channels with GPM as described. After static assembly, biochips were connected to bidirectional perfusion in both channels with 50 μL/min matching shear stress rates of 0.013 dyn/cm^2^ (0.0013 Pa) in the top channel and 0.006 dyn/cm^2^ (0.0006 Pa) in the bottom channel. Gut models were pre-perfused for 5 days prior to triple-model connection with medium exchange every two to 3 days.

Both the liver and placenta models were perfused in channels with LSECs and MDMs (liver) or BeWo (placenta) with VPM at 25 μL/min (0.0065 dyn/cm^2^, 0.00065 Pa). Placenta models were pre-perfused for 24 h prior to triple-model connection. Liver models were not pre-perfused prior to triple-model connection.

**FIGURE 1:**
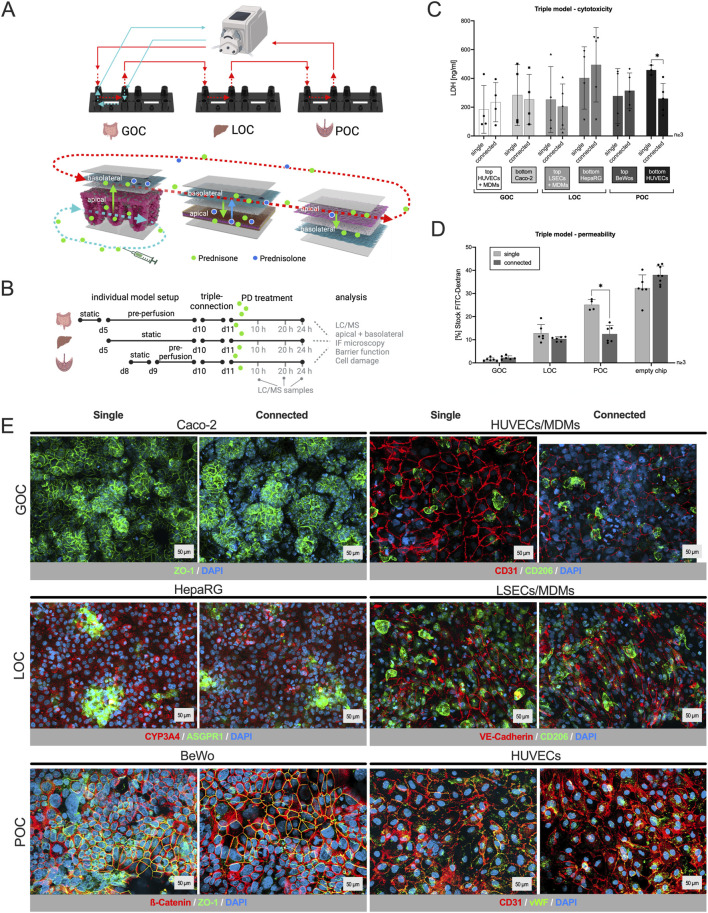
Human three-organ model from gut, liver and placenta retains characteristic features, viability and barrier integrity. **(A)**/top: Schematic of the interconnected three-organ model via tubings and a peristaltic pump (created with BioRender.com); bottom: Distribution pathway of the drug in all three organs: apical drug application in the gut model (bottom channel), transport across the barrier and transfer via perfusion in the upper channel of each model, metabolization of the drug in the hepatocyte channel (bottom) of the liver model, and potential transplacental transfer of the drug and its metabolite into the fetal compartment (bottom). **(B)** Time schedule for the assembly of the individual organs, triple connection, prednisone treatment (PD) and sample collection. **(C)** LDH quantification in supernatants 24 h after the triple connection or single perfusion from all six channels of the three-organ model. The comparison with single models showed that the connection of the organ models has no influence on their viability. Data shown are mean ± S. D; n ≥ 3; 2-tailed unpaired t-test with GOC_top: t = 0.4726, df = 5.783; GOC_bottom: t = 0.2178, df = 5.779; LOC_top: t = 0.3613, df = 5.123; LOC_bottom: t = 0.5775, df = 6.955; POC_top: t = 0.3356, df = 4.908; POC_bottom: t = 3.887 df = 5.199; p (*) < 0.05. **(D)** Barrier function assay using fluorescein-isothiocyanate-labeled (FITC) dextran proving that the connection of three organ models has no influence on the cell barrier of the models. Data shown are mean ± S. D; n ≥ 3; 2-tailed unpaired t-test with GOC: t = 1.775, df = 9.759; LOC: t = 1.534, df = 5.631; POC: t = 6.599, df = 7.999; p (*) < 0.001. **(E)** Immunostaining of the individual cell layers of the different organ models, cultivated either singly or connected for 48 h. DAPI counterstaining (dark blue) in all pictures; cell type-specific markers: GOC (Caco-2) – ZO-1 (green); GOC (HUVECs/MDMs) – CD31 (red), CD206 (green); LOC (HepaRG) – ASGPR1 (green), CYP3A4 (red); LOC (LSECs/MDMs) – VE-Cadherin (red), CD206 (green); POC (BeWo) – β-Catenin (red), ZO-1 (green); POC (HUVECs) – CD31 (red), von Willebrand Factor (vWF, green). Scale bar = 50 μm.

Medium was exchanged in all channels and reservoirs before triple-model connection. Using silicon tubing, the top channel of the gut model with HUVECs and MDMs was connected with the top channel of the liver model containing LSECs and MDMs and the latter with the top channel of the placenta model with BeWo cells creating a cyclic flow, which simulates the physiological blood circulation (Figure 1A). Top channels were perfused at a rate of 25 μL/min, while the gut Caco-2 channel was perfused at a rate of 50 μL/min. The bottom channels of the liver and placenta model were maintained in a static state.

### 2.8 Drug application

Prednisone (CAS: 53-03-2, Merck) was dissolved in DMSO to obtain a 50 mM stock solution. The stock solution was diluted in VPM or GPM to the respective working concentrations, not exceeding a DMSO concentration of 0.1% to avoid non-specific solvent toxicity. Diluted prednisone was administered at a concentration of 55 nM in the Caco-2 channel of the single-gut model, the LSECs channel of the single-liver model and the BeWo channel of the single-placenta model. To achieve a prednisone concentration of 55 nM in the triple model, it was administered at a concentration of 142 nM in the Caco-2 channel (gut model), taking into account the dilution by the additional volume of the liver and placenta compartments. This increase in concentration corresponds to the increase in volume in the triple model compared to the single model. Prednisone administration was initiated 24 h post triple-model connection for 24 h. Control chips for single and connected perfusion were left untreated and were run with medium containing 0.1% DMSO. Supernatants of 80 µL were collected directly from the reservoirs of perfused channels or medium exchange of statically cultured channels after 10 h, 20 h, and 24 h of treatment for downstream analysis of lactate dehydrogenase (LDH) and for HPLC-MS/MS measurements. HPLC-MS/MS samples were directly stored at −20°C, while samples for LDH analysis were stored at 2°C–8°C until measurement.

### 2.9 Drug adsorption

Drug adsorption to the biochip periphery was assessed in cell-free biochip setups. Different concentrations of prednisone (5.5 nM, 55 nM and 550 nM) in supplemented ECGM MV with AAS were transferred to the single setups as well as to the triple setup, filling the total volume of the system, including reservoirs, tubing, and top and bottom channels of the biochip. Samples were collected after 24 h of drug perfusion and stored at −20°C until HPLC-MS/MS analysis. Samples from each compartment of the triple model were pooled before storing.

### 2.10 Immunofluorescence staining

All steps were performed using PBS containing Ca^2+^/Mg^2+^ (Capricorn). Membranes were excised from the biochips using a scalpel and transferred into a PBS-loaded 24-well plate. Membranes were washed once with PBS and fixed with either Histofix 4% (Carl Roth, Karlsruhe, Germany) for 15 min at RT or ice-cold methanol (Carl Roth) for 15 min at −20°C. After fixation, membranes were washed with PBS and incubated in permeabilizing/blocking solution containing PBS with 0.1% saponin (Carl Roth) and 3% normal donkey serum (NDS, Abcam, Amsterdam, Netherlands) for 30 min at RT. Upon permeabilization and blocking, membranes were incubated in permeabilizing/blocking solution containing diluted primary antibody (Supplementary Table S1) at 2°C–8°C overnight. Stained membranes were washed in PBS with 0.1% saponin and incubated in permeabilizing/blocking solution containing diluted secondary antibody (Supplementary Table S1) for 1 h at RT. Membranes were washed again with PBS with 0.1% saponin, PBS, and water and were mounted between two glass coverslips using fluorescent mounting medium (Agilent, Waldbronn, Germany). For the staining of BeWo cells the membranes were fixed with ice-cold methanol for 5 min at −20°C. After washing with fresh PBS, the membranes were incubated with a blocking solution (0.1% FCS-PBS) for 20 min at room temperature. After blocking, the membranes were incubated with fluorophore-coupled antibodies (Supplementary Table S1) for 2 h at 37°C with humidity. Nuclei were stained with 1 μg/mL of 40,6-Diamidine-20-phenylindole dihydrochloride (DAPI, Life Technologies, Karlsruhe, Germany).

### 2.11 Image acquisition and analysis

Fluorescence images were acquired as Z-stacks using the AxioObserver Z1 fluorescence microscope with the ApoTome-2 (Carl Zeiss AG, Jena, Germany) and the AxioObserver 7 fluorescence microscope with the ApoTome-3 (Carl Zeiss AG). All images were taken with a PlanApochromat 20x/0.8 M27 objective (Carl Zeiss AG). Exposure times were set analogues for all images. The ZEN 2 Pro software was used for manual control of the microscope, image acquisition and image compression using the ApoTome Raw Convert function. ZEN 3.5 (blue edition) software was used for subsequent orthogonal projection and image processing.

### 2.12 Permeability assay

Fluorescein isothiocyanate–dextran (FITC-dextran, CAS: 60842-46-8, Merck) with an average molecular weight of 3–5 kDa was used. Medium in both channels was replaced by pre-warmed phenol red-free William’s Medium E. A volume of 250 µL 1 mg/mL FITC-dextran solution in DMEM without phenol red was added to the gut top channel. This step was repeated to minimize dilution of the FITC-dextran solution within the channel. The biochips were incubated for 1 h at 37 C and 5% CO_2_ and protected from light. The 40 kDa FITC-dextran was used to test the POC model. Similarly, 250 µL (1 mg/mL) of FITC-dextran solution was added to the maternal top channel and the biochips were incubated for 30 min at 37°C and 5% CO_2_ and protected from light. Both solutions in top and bottom channel were collected and transferred to a black 96-well microplate (Corning Incorporated, New York, USA). Fluorescence (wavelengths: excitation 492 nm, emission 518 nm) was measured in a microplate reader (INFINITE 200 PRO, Tecan). To avoid values over measuring range, automatic range finder was selected. FITC-dextran concentrations were calculated from a standard curve obtained from the 1 mg/mL stock solution.

### 2.13 Measurement of cytotoxicity (LDH)

LDH concentrations were measured in medium supernatants using the Cytotoxicity Detection Kit PLUS (Roche, Basel, Switzerland). In the case of 2D assays, cell culture plates were centrifuged at 250 g for 10 min at RT to remove cell debris. Supernatants were diluted 1:2 in PBS prior to the assay. The assay was performed as described in the protocol provided by the manufacturer. Absorption was measured at 490 nm with a reference wavelength of 620 nm in a microplate reader (INFINITE 200 PRO, Tecan). LDH concentrations were calculated from a LDH standard (Merck) curve.

### 2.14 HPLC-MS/MS measurements

Samples were analyzed with HPLC-MS/MS via direct injection as formate aducts by JenaBios GmbH, Jena, Germany.

#### 2.14.1 Sample preparation

Samples (100 µL) were spiked with 10 ng internal standard (prednisolone-9,11,12,12-d4 (Merck) and vortexed for 30 s. No further clean-up was performed. Samples were placed into an autosampler and injected directly (5 µL). For quality control blanks of matrix solutions as well as spikes (50 ng/mL) were analyzed. Furthermore, control samples were cross-checked. Sample concentrations above 100 ng/mL were diluted with matrix solution.

Stock solutions of prednisone and prednisolone (1 mg/mL in DMSO USP standards (Merck)) were diluted with pure matrix solution to concentrations appropriate for calibration (100, 10, 1 ng/mL).

#### 2.14.2 HPLC-MS/MS conditions

Chromatographic separation was performed on a Shimadzu HPLC-XR system (Shimadzu Deutschland GmbH, Duisburg) consisting of LC-20AD pumps, PAL autosampler PAL HTC-xt, degassing units DGU-405 and DGA-20A_5R_, oven CTO-20AC and controller CBM-20A. Separation was achieved with a Gemini 5 µm C18 110A; 150 × 3 mm column (Phenomenex, Aschaffenburg, Germany). Oven Temperature was 35°C. The mobile phase consisted of (A) 0.1% formic acid (puriss.p.a. (Merck)) and 5 mM ammonium formate (for mass spectrometry (Merck)) in water (ChemSOLUTE for LC-MS, Th. Geyer, Renningen, Germany) and (B) acetonitrile (ChemSOLUTE gradient grade for HPLC, Th. Geyer Renningen), delivered at a flow rate of 0.40 mL/min. The binary gradient elution program was as follows: 0–16 min, 0%–100% B; 16–23 min, 100% B isocratic; 23–24 min, 100%–0% B; 31 min stop. The injection volume was 5 µL (Table 1).

**TABLE 1:** MRM.

	Q1	Q2	DP	EP	CE	CXP	RT min	LOQ (ng/mL, 5 µL)
Prednisone 1	403.3	299.1	−50	−10	−25	−23	10.9	2
Prednisone 2	403.3	285.1	−50	−10	−40	−23	10.9	4
Prednisolone 1	405.2	295.0	−50	−10	−42	−23	10.8	2
Prednisolone 2	405.2	280.0	−50	−10	−48	−23	10.8	3
Prednisolone D4-1	409.2	333.0	−50	−10	−25	−23	10.8	
Prednisolone D4-2	409.2	317.0	−50	−10	−35	−23	10.8	

Mass spectrometric detection was carried out using an AB Sciex Triple Quad 5500 (Darmstadt, Germany) with Analyst 4.1 software. The ionization mode was negative electrospray ionization (ESI) of the analyte-formate aducts. The MS parameters were set as follows: curtain gas (nitrogen) temperature 400°C, curtain gas pressure 40 psi; ion spray voltage −4500 V; collision gas pressure 8 psi. Data acquisition was performed in multi reaction monitoring (MRM) mode, see table.

#### 2.14.3 Data analysis

Sciex Analyst 4.1 software was used for data acquisition and processing. Calibrations were constructed using linear regression of peak areas versus concentration. Calibration points were set in the linear range between 1–100 ng/mL. The method was validated for selectivity, linearity, matrix effect, accuracy and precision according to DEV guidelines for water and solid examinations.

### 2.15 Statistical analysis

All reported experiments were performed at least 3 times, with at least 2 technical repeats in each group. Data is represented as mean ± S.D. Graph plotting and statistical analysis of biological readouts was performed on GraphPad Prism v10.3.0 (GraphPad Software, La Jolla, CA, USA). Statistical significance for 2 group comparisons were determined using two-tailed Student’s t-test, using Welch’s correction for unpaired t-test. For multiple comparisons comparing to a single control group, one way ANOVA was used with Dunnett’s multiple comparison test. All n, p-values and F, df values are mentioned in the respective figure or the associated legends.

### 2.16 Development of MPS digital twins

Our approach maps the chip architecture to a compartmental model to describe the time-dependent distribution of a compound on-chip. The compartment model uses time-dependent ordinary differential equations and assume well-mixing within compartments. These equations are generally accepted to describe the distribution of exogenous and endogenous compounds and molecules (Aravindakshan et al., 2025). A physical chamber separated by a membrane or connected by flow to another chamber is represented by a compartment in the software. Serial compartments are connected via concentration-dependent flow rates (typically in mL/min) between the compartments and normalized by the volume of the originating compartment. Movement across a membrane is described by permeation and the corresponding surface area. To predict the PK of prednisone and its main metabolite prednisolone on-chip, a digital twin of each of the individual MPS was developed. Clearance of prednisone (liver) and production of prednisolone (liver) were estimated by least-square fitting of model parameters to the observed PK data. For gut and placenta-chips, the permeability across the tissue barrier was estimated to best describe the PK data. The least-square approach was implemented in R and minimized the squared weighted difference (ssq) between the model prediction (pred) and the experimental observation (obs) (Equation 1):

$$ssq=\min\sum \frac{pred-obs}{obs}$$ (1)

Following, a digital twin of the 3-way interactome was developed (Figure 6). Here, the distribution after ‘oral’ administration of prednisone was predicted using the key PK-related parameters (clearance, metabolite formation, permeability across tissue barrier) from the single MPS experiments.

### 2.17 Development of human digital twins

To predict the human PK of prednisone, first, a PBPK was developed using qualified installations of the PBPK software PK-Sim (Frechen et al., 2021). A whole-body PBPK model (Supplementary Figure 2.1) includes an explicit representation of the organs most relevant to the uptake, distribution, excretion, and metabolism of the drug. These typically include the heart, lungs, brain, stomach, spleen, pancreas, intestine, liver, kidney, gonads, thymus, adipose tissue, muscles, bones, and skin. More information can be found in the supplementary material (Supplementary Figure 2.2).

The tissues are interconnected by arterial and venous blood compartments, and each is characterized by an associated blood flow rate, volume, tissue partition coefficient, and permeability (Kuepfer et al., 2016). The analytical approach is based on the principles set out in the guidelines of the EMA, FDA, and/or OECD for reporting on PBPK M&S (EMA, 2024).

The developed PBPK model is used to describe the human kinetics of prednisone. Key kinetic parameters are informed by either clinical data, literature values or on-chip predictions (Table 2). In short, fraction unbound in plasma of prednisone was reported to be around 0.29 (van Runnard Heimel et al., 2005), lipophilicity at 1.5, total liver clearance was reported to be 3.2 mL/min/kg in non-pregnant and around 9 mL/min/kg in pregnant women (Ryu et al., 2018) both at 10 mg single dose, and renal clearance was neglected at 5% (Rose et al., 1981), (Jusko, 2013); solubility was 0.1 mg/mL. Clinical PK data were digitized from (Ryu et al., 2018), (Xu et al., 2007).

**TABLE 2:** Overview of compound characteristics and digital twin model performance.

Parameter	Source	Prednisone	Prednisolone	References
Lipophilicity [a.u]	Literature	1.50	1.66	Dallmann et al. (2018a), Maass et al. (2023)
Molecular weight [g/moL]	Literature	358.43	360.44	
Plasma Cmax [nM]	Literature	55.0	400.0	
Simulated Fetal Cmax [nM]	Simulation	36.0	62.0	N/A
FM^a^ [a.u.]	Literature	1.6 (0.9–2.5)	0.12	van Runnard Heimel et al. (2005)
	Simulation	0.45	0.17	N/A
	MPS	1.3	N/A	
Clearance [mL/min/kg]	Literature	9.0	9.0	Frey and Frey (1990)
	Simulation	10.1	5.0	N/A
	MPS	12.4	1.9	
Permeability [E−6 cm/min]	Literature	22	N/A	Paixão et al. (2010)
	Simulation	4.2		N/A
	MPS	2.9		

^a^ FM, fetal/maternal ratio.

### 2.18 Pregnancy model

In addition to the base PBPK model, a pregnancy (fetal-maternal) PBPK model was implemented as outlined before (Dallmann et al., 2018a) which is publicly available, and used to describe the systemic exposure of prednisone in the fetus. For a more detailed description of the implementation workflow within PK-Sim^®^ and MoBi, we refer to the tutorial (Dallmann et al., 2018a). Reported fetal/maternal plasma ratio of prednisone is 1.67 (van Runnard Heimel et al., 2005). In short, literature, fitted, and MPS-based PK-related parameters were implemented in the pregnancy PBPK model and partition coefficients from main plasma to placenta were adjusted to match observed plasma ratios. The parameter combinations were then used to simulate maternal and fetal prednisone kinetics in n = 100 virtual women for up to 3 months (100 days).

## 3 Results

### 3.1 Connecting single-organ models to form the three-organ system does not compromise their viability or barrier integrity

The centerpiece of this study is a dynamic and physiologically relevant MPS that integrates the gut, liver, and placenta to investigate drug absorption, distribution, and metabolism. An MPS using Caco-2 cells (intestinal epithelial cells, grown hanging in bottom channel), HUVECs (human umbilical vein endothelial cells, grown together with MDMs in top channel), and MDMs (monocyte-derived macrophages) replicates the intestinal barrier’s microenvironment (Blanford and Murphy, 1977; Shroff et al., 2022). Caco-2 cells form an intestinal epithelium with villus-like structures under perfusion (Kim and Ingber, 2013), allowing the study of drug absorption and first pass metabolism. HUVECs simulate the endothelial barrier of blood vessels, facilitating studies on vascular permeability and MDMs are incorporated to represent immune cells, enabling the investigation of immune responses and inflammation. They further regulate tissue homeostasis by improving enzyme expression and barrier functionality in OoC models (Rennert et al., 2015). The liver model with HepaRG (hepatocytes, grown hanging in bottom channel), LSECs (liver sinusoidal endothelial cells, grown together with MDMs in top channel), and MDMs simulate the liver’s microenvironment (Rennert et al., 2015), (Gröger et al., 2016). It enables the study of hepatic functions like drug metabolism. The placenta is modeled using the human trophoblastic BeWo cells (grown in top channel) and HUVECs (grown hanging in bottom channel) to replicate the maternal-fetal interface. BeWo cells are the second most used cell line that retains the key features of trophoblast cells allowing the study of drug transfer across the placental barrier (Pastuschek et al., 2021). It provides a realistic platform for understanding placental physiology and how drugs are processed and transported in pregnant women.

Each model was established separately through sequential cell seeding and pre-perfusion for the gut model, before connecting them via perfusion and drug application the day after (Figure 1B). To ensure the robustness and functionality of the connected three-organ model and to rule out impairments caused by the presence of the other organ models, we first assessed the viability, barrier integrity and marker expression of each individual organ model in comparison to the connected perfusion set up.

The viability of the models was evaluated using an LDH cytotoxicity assay. Only upon cell damage the intracellular LDH (lactate dehydrogenase) can be measured in perfusates. Apical and basolateral compartments of each organ, singly perfused or connected, were monitored individually (Figure 1C). LDH values vary normally between the biological replicates of the experiment, but no difference was measured within an experiment when models were cultured singly or in interconnection. Overall, the integrated gut-liver-placenta MPS maintained high cellular viability across all culture conditions.

The barrier integrity of the gut, liver and placenta models was measured by a fluorescein-isothiocyanate-labeled (FITC) dextran permeability assay (Figure 1D). When models were connected, permeability remained the same or tended to decrease (POC), which confirms that the process of connecting these organ chips did not adversely affect their barrier functions.

Similarly, immunostaining (Figure 1E) did not show any effect on cell integrity or marker expression when looking at the individual cell layers of each organ after single or connected perfusion.

These results validate the stability and functionality of our integrated MPS platform, providing a reliable foundation for further pharmacokinetic and pharmacodynamic studies.

### 3.2 The three-organ model of gut, liver, and placenta maintained their cellular and barrier integrity during treatment with prednisone

As mentioned in the introduction, prednisone was used as a model drug in this study. It was designed to mimic the effects of cortisol, a hormone that regulates inflammation, immune response, and metabolism in the body. Prednisone is commonly used in medical treatments for its anti-inflammatory and immunosuppressive properties (Chaphekar et al., 2021), (Corticosteroids, 2012). Understanding its pharmacokinetics—how it is taken up, distributed, and metabolized in the human body—is crucial for its effective clinical use. After confirming that the connection of the three organ models did not affect their integrity, the next step was the administration of prednisone to the luminal side of the gut model 24 h after starting triple-perfusion. Preliminary experiments showed that the treatment of all relevant cell types with prednisone in a concentration range of 0.5 nM–50 µM did not result in any damage or activation of the immune cells (Supplementary Figure S1). Hence, since prednisone was not expected to be harmful to any of the tissues, we monitored the model integrity by assessing cell viability/cytotoxicity, barrier function, and performing immunostaining for tissue markers.

**FIGURE 2:**
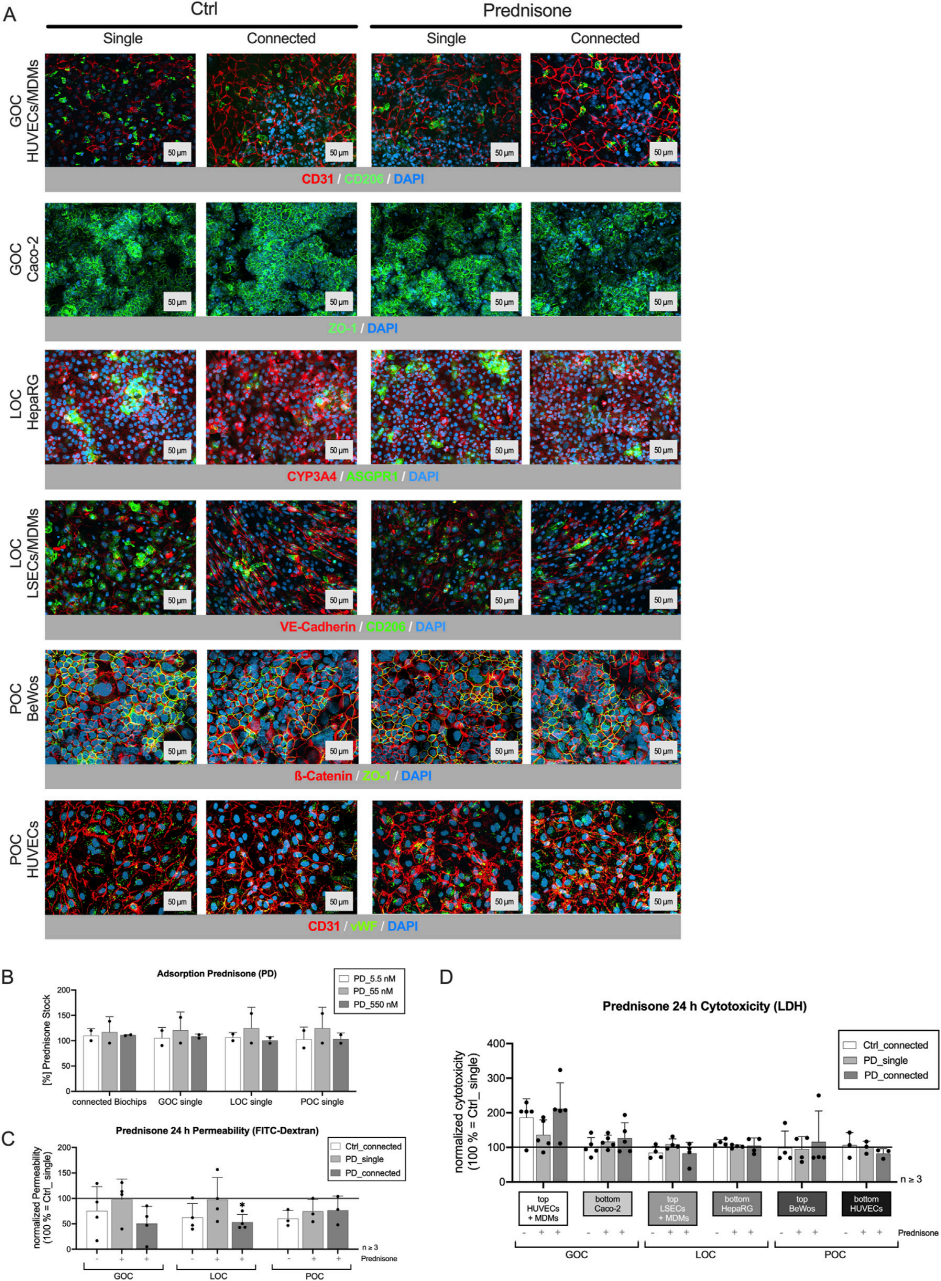
Prednisone (PD) treatment did not affect cell viability, tissue integrity or barrier function of the three-organ model. **(A)** Immunostaining of the individual cell layers of the different organ models, cultivated either individually or connected for 24 h, followed by a 24 h incubation with medium (Ctrl) or PD (55 nM single, 142 nM connected). DAPI counterstaining (dark blue) in all pictures; cell type-specific markers: GOC (HUVECs/MDMs) – CD31 (red), CD206 (green); GOC (Caco-2) – ZO-1 (green); LOC (HepaRG) – ASGPR1 (green), CYP3A4 (red); LOC (LSECs/MDMs) – VE-Cadherin (red), CD206 (green); POC (BeWo) – b-Catenin (red), ZO-1 (green); POC (HUVECs) – von Willebrand Factor (vWF, green), CD31 (red). Scale bar = 50 μm **(B)**: Binding properties of PD to chip and perfusion material, three different concentrations (5.5, 55, 550 nM, dissolved in cell culture medium) were perfused for 24 h in a cell-free system. Adsorption rate (%) was determined by comparing PD concentration before (stock solution) and after perfusion (via HPLC-MS/MS). No significant adsorption was measured. Data shown are mean ± S. D; n = 2; one-way ANOVA, all conditions DF = 3; Connected: F = 0.4817, GOC_single: F = 0.5758, LOC_single: F = 0.7867, POC_single: F = 0.8170, p < 0.05. **(C)** Compared to untreated single-perfused chips, the barrier function test with fluorescein isothiocyanate-labeled (FITC) dextran shows that the combination of the three organ models as well as the treatment with PD does not damage the cell barriers. Data shown are mean ± S. D; n ≥ 3; one-way ANOVA, all conditions DF = 3; GOC: F = 3.656, LOC: F = 4.176, POC: F = 2.094, p (*) < 0.05 **(D)** LDH quantification in supernatants 48 h after triple connection and 24 h after PD administration showed similar values compared to the untreated single models and excludes cytotoxicity upon PD treatment; samples were taken from all six channels of the three-organ model. Data shown are mean ± S. D; n ≥ 3; one-way ANOVA, all conditions DF = 3; GOC_top: F = 7.184, GOC_bottom: F = 1.265, LOC_top: F = 2.189, LOC_bottom: F = 1.167, POC_top: F = 0.1972, POC_bottom: F 1.287; p < 0.05.

Immunostainings (Figure 2A) revealed no changes in cell integrity and marker expression, irrespective of whether the organ models were perfused individually or connected in series, nor did the treatment with prednisone induce any noticeable alterations.

Compared to untreated, single-perfused control setup, the permeability assay (Figure 2C) showed that the barriers remained intact or even improved under all tested conditions. Additionally, LDH measurements in the perfusates (Figure 2D) indicated that prednisone treatment did not affect cell viability in any of the MPS setups. The higher variability observed in samples from the gut model is anticipated due to the high cell shedding rates characteristic of gut epithelial tissue.

Thus, the distribution of prednisone and its metabolite, prednisolone, throughout the entire three-organ system occurred across intact cellular barriers. Any influence from compromised barriers or technical difficulties can be excluded. This is crucial for the reliability of the system and the interpretability of the PK/PD data.

Furthermore, the measurement of three prednisone concentrations before and after perfusion within the cell-free triple setup and all cell-free single setups (Figure 2B) revealed no significant binding of the drug to the plastic surface of the biochip or the perfusion equipment. Measuring drug adsorption to the biochip periphery is particularly relevant for prednisone. Due to its hydrophobic nature, prednisone is prone to adsorption onto the materials of commonly used in biochips. This can result in lower drug concentrations, potentially leading to underestimation of drug effects or efficacy.

Additionally, no binding to serum components of the cell culture medium was detected (data not shown). Therefore, it can be assumed that the intended concentration of 55 nM (Frey and Frey, 1990) (corresponds to the C_max_, the highest concentration of a drug in blood plasma that can be reached after administration) is available to the cells.

### 3.3 Distribution of prednisone and its metabolite prednisolone over 24 h after oral administration

**FIGURE 3:**
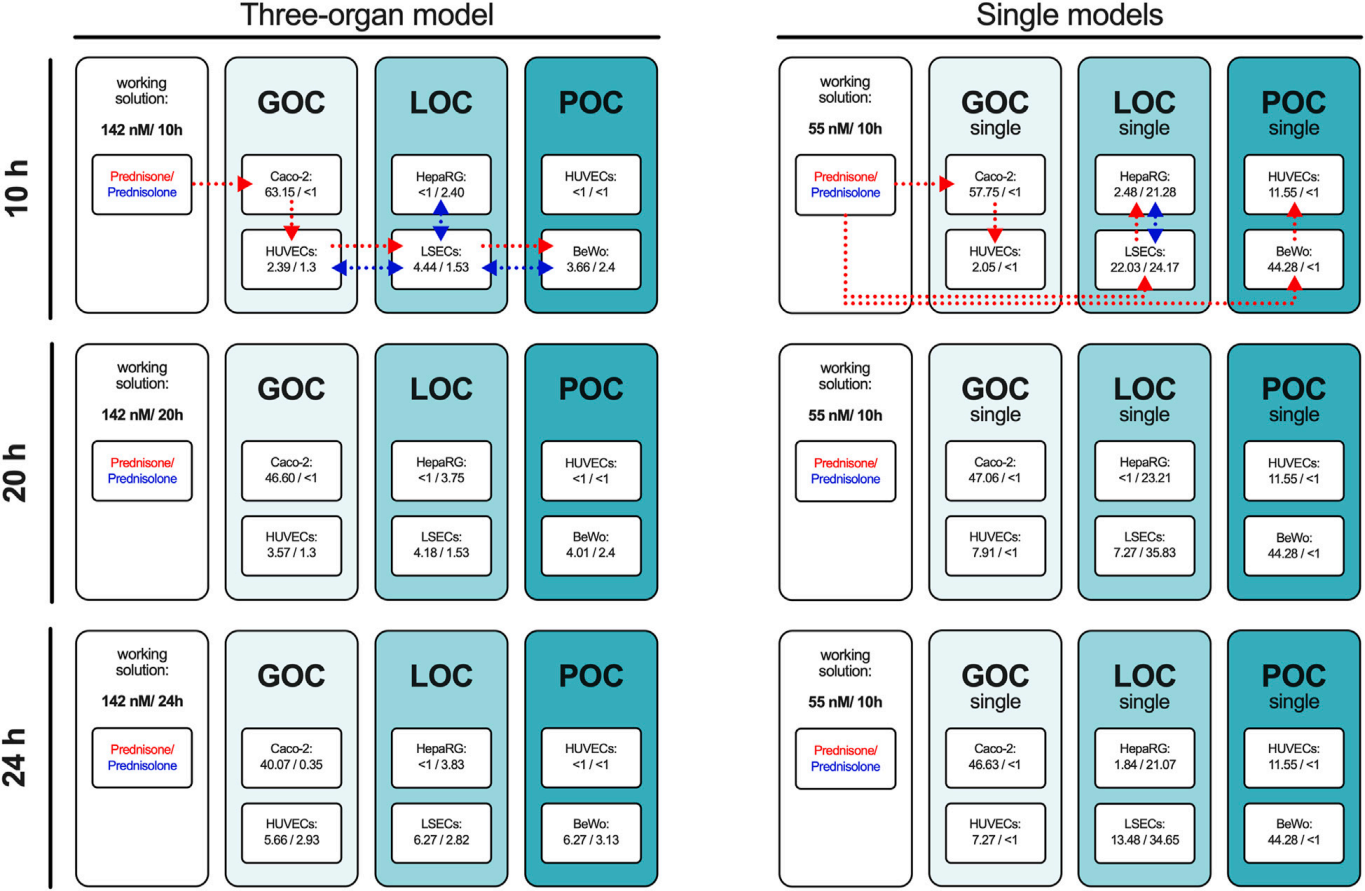
The percentage of prednisone and prednisolone in relation to the set working concentration in the individual compartments of the three-organ model and in the individual perfused models. The concentrations of prednisone and prednisolone were measured in the supernatants of all compartments of the 3-organ model and all single models after 10, 20, and 24 h using HPLC-MS/MS. The percentage values relative to the administered concentration of prednisone are presented as the mean of four independent experiments. Dotted lines with arrowhead indicate drug administration and distribution routes for prednisone (red) and prednisolone (blue) in the three-organ model and single organ models (representative scheme applies to all measured time points).

The prednisone distribution dynamics across the different compartments of the three-organ model as well as of single-perfused models were analyzed by sampling the supernatant at 10, 20, and 24 h, with its concentration and metabolization to prednisolone measured via HPLC-MS/MS (Figure 1B). Due to the higher medium volume in the triple system compared to single-perfused models with a constant volume at substance application, the concentration of prednisone was increased from 55 nM applied in single models to 142 nM in the triple model. The percentage distribution of prednisone and prednisolone in the individual compartments of the models is shown over time in Figure 3, the corresponding concentrations of each experiment are listed in Supplementary Table S2.

In the connected model prednisone was administered orally on the luminal side of the gut model. Ten hours after administration, 63% of the initial prednisone was still present in the gut compartment. A fraction of the prednisone had entered the circulation, and hepatic metabolism had already begun converting prednisone to prednisolone.

After an additional 10 h, the circulating prednisone levels had slightly increased, and the amount of prednisolone in the hepatic compartment had also risen, with prednisolone being transferred into the circulation.

Twenty-four hours post-oral administration, the prednisone level in the gut lumen further decreased, while its circulating levels continued to rise, along with the amount of prednisolone. However, prednisolone did not transfer into the fetal compartment of the placenta model and was only detected in minor quantities on the luminal side of the gut.

The single-gut model displayed similar kinetics in prednisone transfer from the apical to basolateral side without metabolism to prednisolone. Metabolism was exclusively detected in the liver model, where prednisone was applied on the basolateral side of the liver sinusoidal endothelial cells (LSECs). Only 22% of the initial prednisone was detected after 10 h, with further reduction over time. In the hepatocyte compartment, approximately one-quarter of the initial prednisone was metabolized to prednisolone, with another quarter found in the vascular area of the LSECs, increasing to 35% with longer incubation.

In the single-placenta model, prednisone was applied to the maternal side of BeWo cells, with the majority remaining in this area and only 10%–12% was detected on the fetal side. Although trophoblast cells can metabolize prednisone, the presence of 11β-hydroxysteroid dehydrogenase (11β-HSD2) is reported to convert prednisolone back to prednisone (Sarkar et al., 2001), (Ince-Askan et al., 2019). As expected, no detectable levels of prednisolone were found in our placenta model, this was observed exclusively in the liver model.

These findings indicate effective metabolism and transport of prednisone in the MPS, with distinct distribution patterns observed across the different compartments over time.

### 3.4 Digital twin simulation of prednisone pharmacokinetics

**FIGURE 4:**
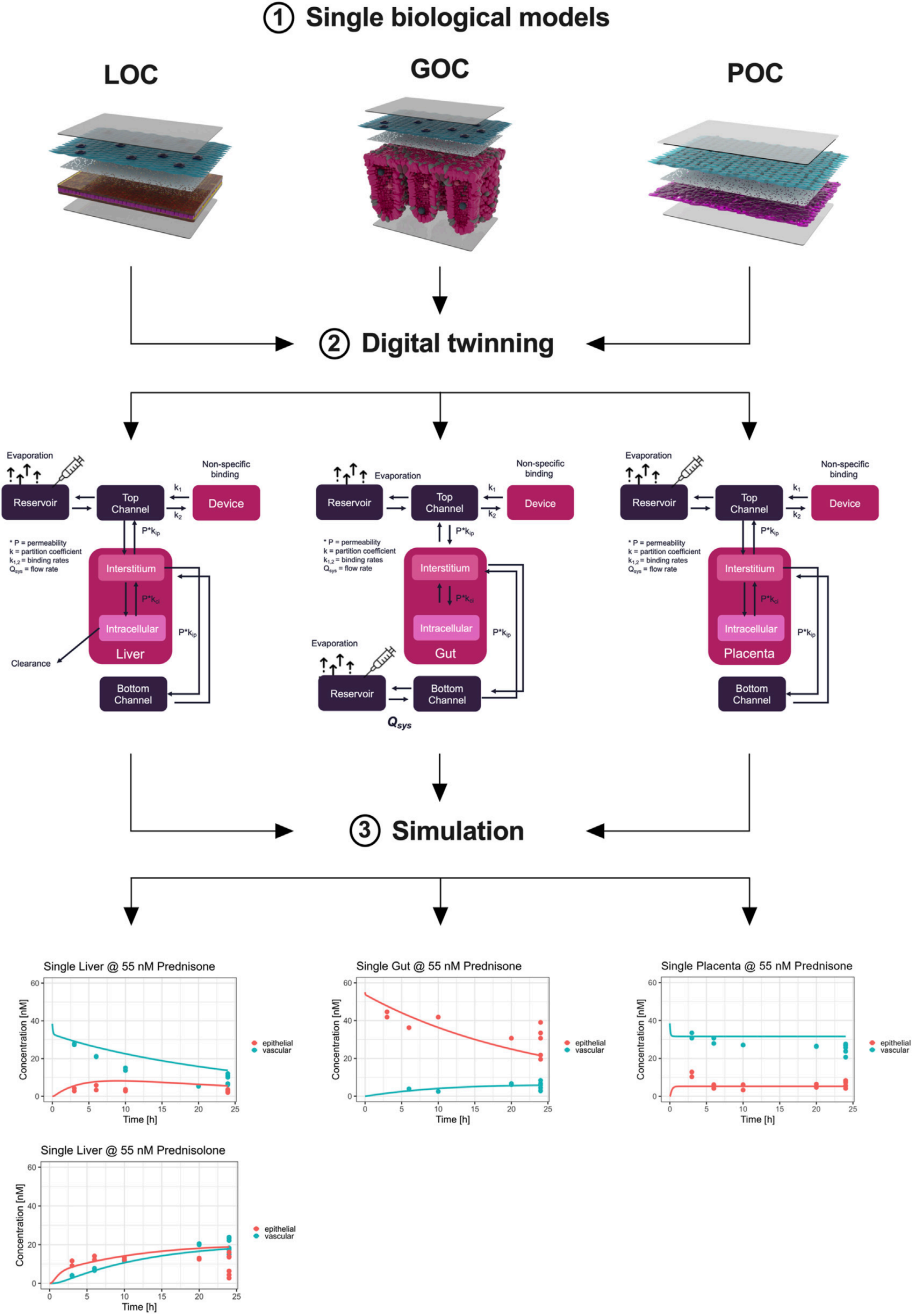
Digital twinning and on-chip pharmacokinetics of prednisone in liver, gut, and placenta models. Information on hardware, the compound, and the biological function (1) investigated are used to inform the development of digital twins (2). Subsequently, the so developed digital twins simulate the distribution of prednisone (3) on-chip by estimating biologically-relevant model parameters such that the model description (lines) matches the observed data (colored dots) as best as possible. The metabolism of prednisone and simultaneous formation of prednisolone was well described by the liver-chip digital twin (lower left). Likewise, the distribution of prednisone in the gut-chip was well captured using the digital twin by estimating absorption rates. Lastly, the placenta-chip showed an intact barrier and efflux properties, as expected, as prednisone is in quick equilibrium after dosing, but at differing concentration levels.

We successfully developed a digital twin to simulate the pharmacokinetics (PK) of prednisone using data derived from the MPS studies. Initially, model parameters were fitted to the observed kinetic data from MPS models representing biological functions—gut absorption, liver metabolism, and placental transfer (Figure 4). The digital twin simulations accurately captured the on-chip pharmacokinetics across all three single MPS.

Subsequently, we integrated these model parameters into the digital twin of the three-organ interactome (gut-liver-placenta). This allowed us to predict the overall pharmacokinetics of prednisone within the interconnected system and evaluate potential organ-organ crosstalk. The digital twin simulations closely aligned with the experimental measurements, though some discrepancies indicated that organ-organ interactions influenced the pharmacokinetics more than expected.

**FIGURE 5:**
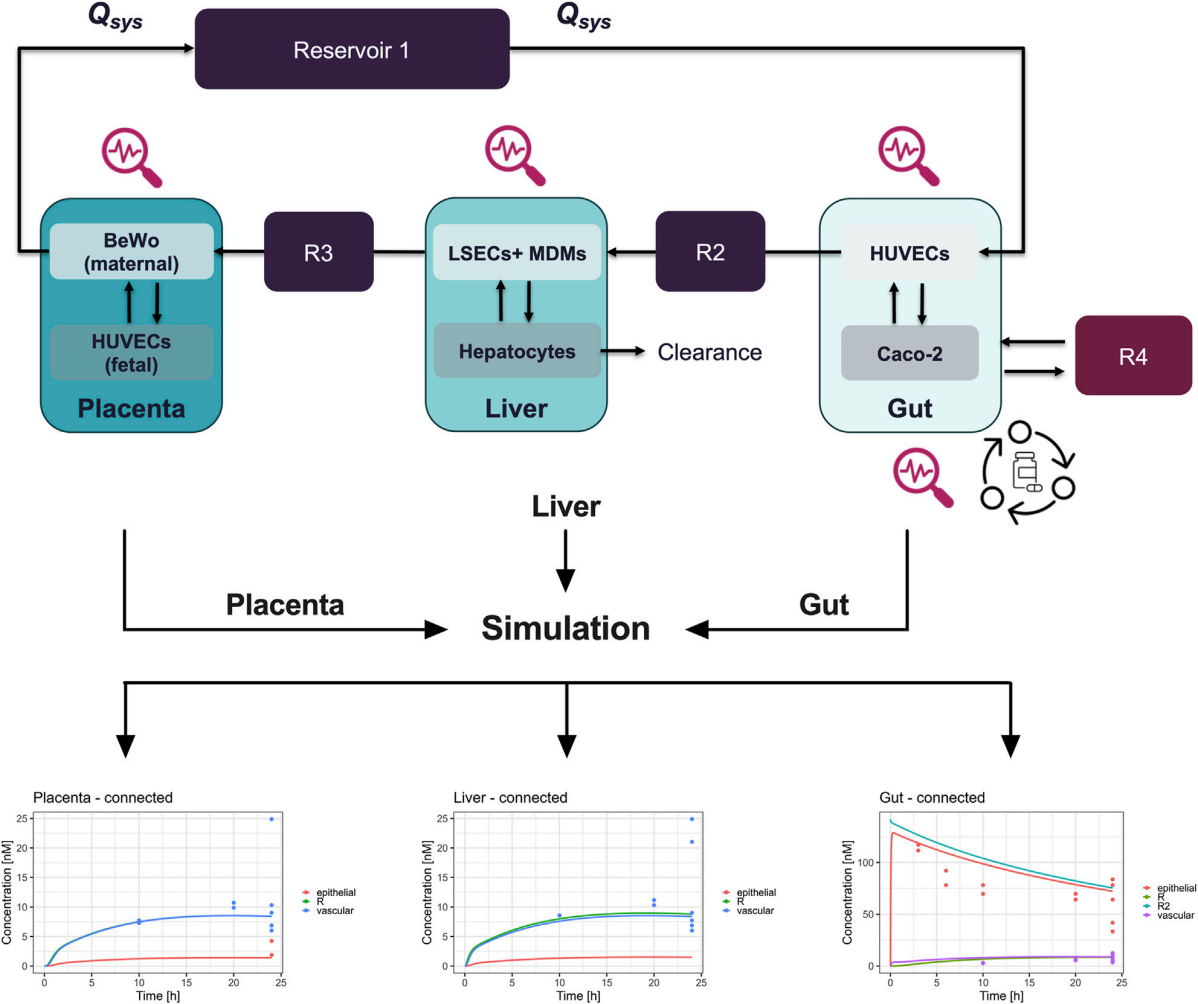
Schematic representation and pharmacokinetics (PK) of prednisone in a connected three-organ microphysiological system (MPS) of gut, liver, and placenta. Top panel: The diagram illustrates the connected three-organ setup, including the gut, liver, and placenta models. Each organ-on-chip model contains cell-specific compartments: Gut (Caco-2, HUVECs, and macrophages), Liver (hepatocytes, LSECs, and macrophages), and Placenta (BeWo cells on the maternal side and HUVECs on the fetal side). Prednisone flows through the system via reservoirs (R2, R3, R4), with clearance occurring in the liver. This setup mimics the physiological transfer of the drug between maternal and fetal compartments. Bottom panels: The pharmacokinetics (PK) of prednisone are shown for each organ under connected conditions over 24 h. Left (Placenta - connected): Concentration profiles of prednisone in the placenta, highlighting drug accumulation in the fetal and maternal compartments. Center (Liver - connected): Prednisone concentration in the liver model, demonstrating significant accumulation in the vascular compartment (blue), with lower levels in the epithelial (red) and reservoir (green) compartments. Right (Gut - connected): Prednisone absorption in the gut model, showing a rapid decrease in the epithelial compartment (red), with a slower transfer to the vascular (purple) and reservoir compartments (green and cyan). These PK profiles reveal the compartmentalized distribution of prednisone across the interconnected gut, liver, and placenta MPS, allowing for a detailed evaluation of drug transfer, metabolism, and clearance in a physiologically relevant system. R = reservoir, R2 = second reservoir.

Following, the model parameters describing the key biological functions were integrated within the digital twin of the 3-way interactome to predict the on-chip kinetics and evaluate the potential for organ-organ crosstalk. When compared to the actual measurements, the 3-way digital twin captured the main kinetics in all three organs well, although some further improvements are possible, indicating that organ-organ crosstalk is changing the PK (Figure 5).

### 3.5 Predicting fetal exposure to prednisone

A whole-body human digital twin was implemented using PK-Sim^®^ to simulate prednisone pharmacokinetics in pregnancy. Initially, this model was qualified using published clinical data for a single 10 mg dose of prednisone. The reported liver clearance (Cl = 9 mL/min/kg) and gut permeability (P = 22E-6 cm/min) resulted in an overprediction of plasma concentrations. After refining these parameters (Cl = 10.1 mL/min/kg; P = 4.2E-6 cm/min), the simulation better matched the observed clinical data (red curve, Supplementary Figure 2.1).

Next, the parameters derived from the single MPS models were translated to human physiology and integrated into the pregnant-woman digital twin (Cl = 12.4 mL/min/kg; P = 2.9E-6 cm/min). The prediction based solely on MPS data produced a good approximation of observed plasma levels in pregnant women. We calculated the fetal/maternal maximum concentration ratio as a quality indicator, with literature reporting a ratio of 1.6 (range 0.9–2.5). Our MPS-derived data yielded a ratio of 1.3, which closely aligns with clinical reports, though the digital twin underpredicted this ratio at 0.5.

### 3.6 Long-term exposure simulations

**FIGURE 6:**
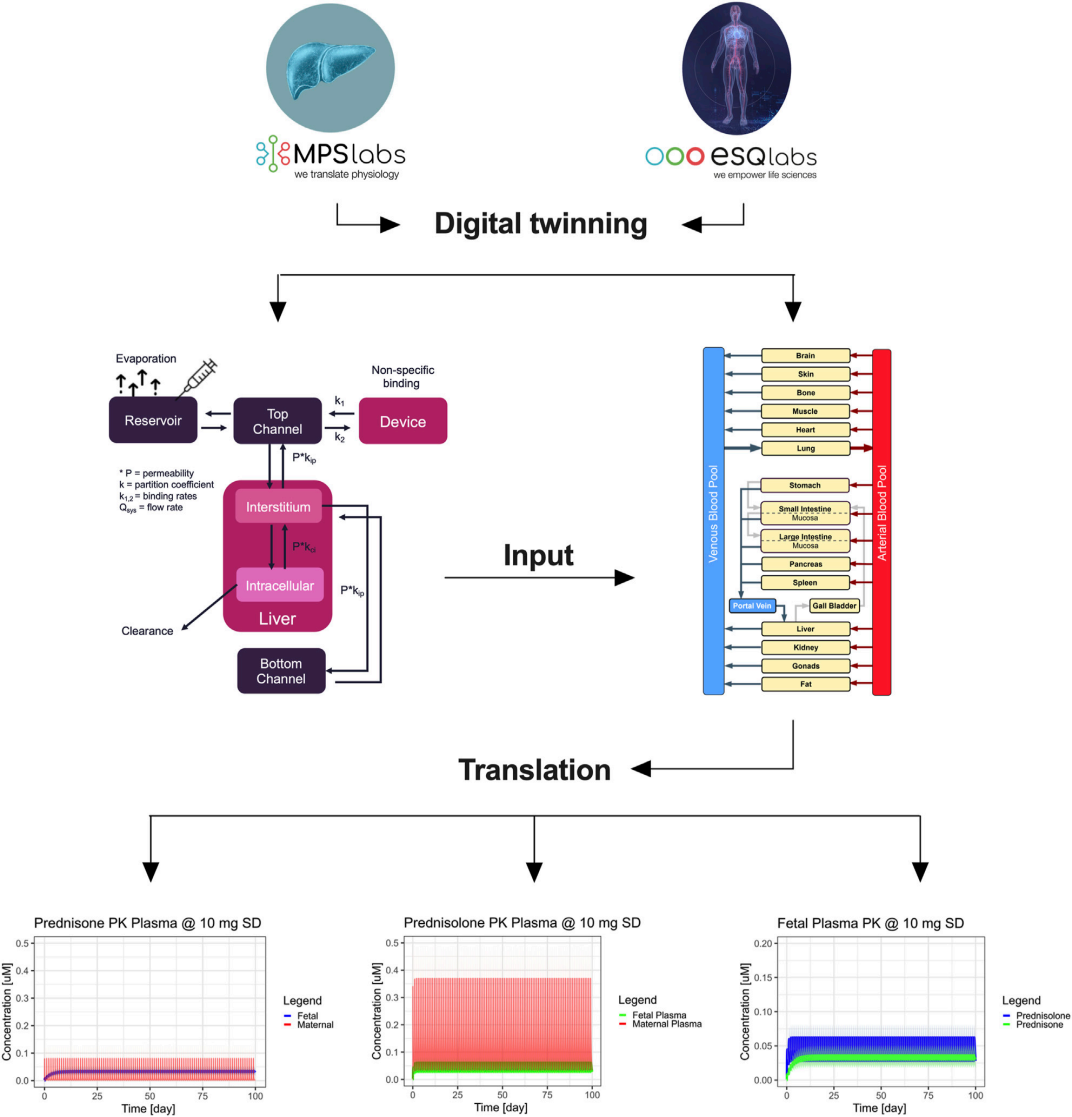
Integration of digital twinning with microphysiological systems (MPS) to predict maternal and fetal pharmacokinetics of prednisone. The workflow depicts the integration of *in vitro* data from MPS models and digital twin simulations to predict the PK of prednisone and prednisolone in pregnant women. At the top, single-organ MPS models (gut, liver, and placenta) are connected to digital twin simulations, which are further integrated into a full-body human digital twin framework. This translation leads to predicting maternal and fetal PK profiles of prednisone and its metabolite prednisolone in pregnant women. The bottom plots represent the concentration-time profiles in maternal and fetal compartments. The right panels illustrate maternal plasma (red) and fetal plasma (blue/green) concentrations of both prednisone and prednisolone over 100 days, showing drug accumulation in the fetal compartment. Shaded areas represent ± 1 standard deviation.

To further investigate fetal exposure, we simulated daily prednisone dosing of 10 mg over 3 months. In the maternal compartment, there was no accumulation of prednisone, with residual levels clearing within 24 h (Figure 6). On the fetal side, prednisone plasma levels showed some accumulation, reaching a steady-state concentration of approximately 38 nM, which remained below the reported clinical maximum of 55 nM. Likewise, the steady-state concentration of prednisolone in the simulated fetus is estimated to be around 47 nM, with a concentration maximum of around 62 nM. This is still an order of magnitude lower than reported averaged plasma concentration maxima of around 400 nM (Table).

These findings suggest that fetal exposure to prednisone and prednisolone is non-toxic at typical therapeutic doses, supporting the utility of this integrated system for early-stage drug safety assessments during pregnancy.

## 4 Discussion

The aims of this work were to integrate three MPS within a single experiment and to present a translational framework to link *in vitro* findings to relevant clinical situations. The presented integrated approach combined biological data from a gut-liver-placenta-MPS and physiology of pregnant women to simulate the pharmacokinetics of prednisone and prednisolone in the fetus. The study established a three-organ model (gut-liver-placenta) using well-known single models connected via perfusion (Kaden et al., 2024), (Maurer et al., 2019), (Kaden et al., 2023a), (Gröger et al., 2016), (Siwczak et al., 2022). Viability, barrier integrity, and marker expression were assessed of each organ model individually and when connected. Viability and barrier integrity remained stable or improved when models were connected, and immunostaining revealed no adverse effects on cell integrity or marker expression in individual cell layers after single or connected perfusion (Figure 1). These results demonstrate the robustness and functionality of the connected three-organ model, ruling out impairments caused by the presence of other organ models. To our knowledge there is no study specifically connecting gut, liver and placenta models, although there are several reports that show the applicability of multi-organ systems in PK analysis (Novak et al., 2020), (Tsamandouras et al., 2017b), (Lee-Montiel et al., 2021), (Edington et al., 2018), (Herland et al., 2020), (Ingber, 2022), (Maschmeyer et al., 2015). To name just two, Vernetti et al. (2017) created a multi-organ chip system that included liver and intestine models, along with kidney, blood-brain-barrier and muscle, showing the potential for connecting gut and liver models (Vernetti et al., 2017). They also showed that the organ-specific processing of trimethylamine and vitamin D3 is consistent with existing clinical data and that trimethylamine-N-oxide crosses the blood-brain barrier. Another study using a 7-way MPS platform demonstrated quantitative PK analysis of diclofenac metabolism that aligned with clinical observation (Edington et al., 2018). Although several groups are actively developing *in vitro* models of the placental barrier to study drug transport (Kammala et al., 2023), (Richardson et al., 2022), (Ghorbanpour et al., 2023), (Elzinga et al., 2023) (and revised in), there remains a significant gap in models that replicate its interconnected nature with other organs.

After confirming that connecting the organ models did not affect their integrity, prednisone a synthetic corticosteroid, was used as a model drug to study pharmacokinetics. Preliminary tests showed no damage or immune cell activation at concentrations of 0.5 nM–50 µM (Supplementary Figure S1). Additionally, no significant drug binding to the biochip surface, perfusion equipment, or serum components was observed, indicating the intended concentration was available to cells (Figure 2). Many biochips are made of polydimethylsiloxane (PDMS), which is known to adsorb small hydrophobic molecules like prednisone (van Meer et al., 2017). Therefore, alternative materials with lower absorption properties, like the ones used in this study, are preferable for testing of hydrophobic molecules. A reduction in the free concentration of the drug can be ruled out, allowing researchers to develop more accurate pharmacokinetic models with enhanced predictive capabilities.

Furthermore, we could exclude that prednisone treatment alters cell integrity, marker expression or barrier function in any setup (Figure 2). The drug distribution occurred across intact cellular barriers, ensuring reliable PK/PD data interpretation.

After 24 h of drug administration, gut prednisone levels decreased, circulating levels of prednisone and prednisolone rose, and only prednisone in single-placenta models did transfer to the fetal placenta compartment. The single-gut model showed similar prednisone transfer without metabolism. Metabolism was exclusively detectable in the liver model.

While prednisone remains a highly effective compound, its main metabolite, prednisolone, has the potential to cross the placental barrier (van Runnard Heimel et al., 2005). Although the placenta provides some protection through enzymatic degradation and selective transfer, after treatment with prednisone, impaired fetal development, and pre-term delivery (Ryu et al., 2018), (Dan et al., 2015), (Cooper et al., 2019) are problems, limiting the use of prednisone. This emphasizes the need for better preclinical models of drug pharmacokinetics.

Maternal drug dosing, fetal exposure, and drug safety in pregnancy are usually studied in post-marketing observational studies. In an earlier phase, non-clinical studies are performed mostly in animals. However, interspecies translation of results is problematic due to differences in placental structure. Therefore, the exploration using human *ex vivo*, *in vitro*, and *in silico* systems may contribute to improving pharmacological and toxicological profiling of drugs administered during pregnancy. For instance, the fetal exposition and pharmacokinetics of several compounds in the human term placental barrier have been studied using the *ex vivo* placenta perfusion system as it closely resembles the human *in vivo* situation (van Hove et al., 2022). However, the technique is expensive, and its success depends entirely on the patient´s clinical health, which is reflected in the tissue integrity. Thus, further standardization of the perfusion technique is needed to facilitate and increase the broader use of perfusion data (Schneider et al., 2022).

In our single placenta model, the percentage of drug transfer from the maternal to the fetal side was approximately 10%–12% over a 24-h period. When compared to the integrated placenta model, the prednisone transfer data (15%–20% observed and simulated) showed an underprediction relative to the clinical scenario.

Animal model data also demonstrated limited alignment with human-based data. For example, mouse models showed a drug transfer rate of approximately 10%, with a maternal-to-fetal transfer ratio of 0.1–0.2 (van Runnard Heimel et al., 2005). Similarly, in human placenta studies using an *ex vivo* placenta perfusion system, the reported transfer percentage of prednisone to the fetal side was less than 10%, with a maternal-to-fetal transfer ratio of 0.1 (Levitz et al., 1978). This trend has also been observed for other tested drugs (Hutson et al., 2011). Our study highlights the potential for an integrated triple-MPS as an advanced *in vitro* tool for the assessment of placental transfer of pharmaceuticals. However, it remains unclear how relevant these findings are within a clinical situation. We, therefore, performed MPS-to-human translation of on-chip pharmacokinetics using a two-stage-approach. First, individual MPS-based PK parameters (Figure 5) were determined by linking the on-chip kinetics of prednisone and prednisolone (liver only) to their respective biological functions. The so estimated liver metabolism of prednisone matched both clinical reports and the optimized PBPK model (MPS: 12.4 mL/min/kg, Lit: 9.1 mL/min/kg (Ryu et al., 2018), Simulation: 10.1 mL/min/kg; Table), while prednisolone was ∼4.7-fold underpredicted (MPS: 1.9 mL/min/kg Lit: 9.0 mL/min/kg (Ryu et al., 2018), Simulation: 5.0 mL/min/kg; Table) when compared to literature, but matched more closely the optimized PBPK model. Prednisone and prednisolone are mainly metabolized by cytochrome P450 enzymes and 11beta-hydroxysteroid dehydrogenase (Frey and Frey, 1990), (Penzak et al., 2005), (Jenkins and Sampson, 1967), (Diederich et al., 2002). Potentially, altered expression levels on-chip may result in slower metabolism of prednisolone. The estimated permeability across the gut-MPS barrier of prednisone (MPS: 2.9E-6 cm/min, Lit: 22E-6 cm/min (Paixão et al., 2010), Simulation: Lit: 4.2E-6 cm/min; Table) showed a slower uptake rate, but matched optimized PBPK model simulations. The reduced metabolic activity of CaCo-2 cells (over human primary cells), as well as cell-associated binding of prednisone to proteins, may likely impact the permeability assessment. The distribution of prednisone in the placenta-MPS resulted in an estimated fetal/maternal concentration ratio that matched clinical reports nicely [1.3 vs. 1.6 (Dallmann et al., 2018a); Table]. Additionally, the placenta-MPS actively showed an efflux ratio (Dallmann et al., 2018b), as indicated by the higher concentration levels in the maternal compartment (Figure 4), confirming its utility in clinical research.

The second stage involved the implementation of the so estimated on-chip PK parameters into the optimized population PBPK model to simulate the distribution of prednisone and prednisolone in pregnant women and the fetus.

We could predict clinically relevant levels of maternal prednisone and likely safe, but underpredicted exposure levels of fetal prednisolone (Figure 6). These findings support the potential of the integrated approach to be applied in prospective drug development. Still, while non-specific binding to chip material and binding to (media) proteins were assessed, quantification of cell-associated or intracellularly bound compounds would not only improve mechanistic detail mapped by the MPS-digital twins, but also further improve the translational predictions. Furthermore, the adoption of the presented workflow would be supported by investigating a broader range of compounds, including those with challenging pharmacokinetics (e.g., prodrugs, drugs with poor solubility, drugs with active metabolites or drugs that interact with multiple receptors or transporters) and that may pose risks to pregnancy and the unborn child. To give just one example that would affect a large number of pregnant women is Caffeine. Caffeine is a stimulant that crosses the placenta more easily than prednisone and can be found in fetal circulation (Temple et al., 2017). The fetus has limited ability to metabolize caffeine due to immature hepatic enzymes. High maternal caffeine intake (typically defined as over 300 mg/day (3 cups of coffee)) has been associated with an increased risk of miscarriage, preterm birth, low birth weight, and potentially impaired fetal development, including neurodevelopmental issues later in childhood (Greenwood et al., 2014). Future validation work with more compounds and drugs will provide further evidence of the utility of this system in predicting human drug behavior and improving our understanding of ADME processes.

Another critical aspect is optimal experimental design for obtaining robust data to determine key PK parameters, such as absorption, distribution, metabolism, and excretion (ADME) (Maass et al., 2019), (Edington et al., 2018), (Nagayasu et al., 2019). In our study, we investigated 54 data points for the liver-MPS, 28 data points for the gut-MPS, and 31 for the placenta-MPS across five time points. This is well in-line with literature reports on liver metabolism and gut permeability, where typically 10–15 data points per parameter, distributed across 3-5 time points and biological replicates, are collected.

To improve the predictive power of these models further, increasing both biological replicates and time points (e.g., four replicates and 6 time points per direction of transport) would provide a more comprehensive dataset (Harrell et al., 1984), (Peduzzi et al., 1996). This results in 3–4 times the data typically gathered in current MPS experiments, reducing uncertainty and considerably improving clinical predictions. However, there are no established guidelines in the literature for standardizing the number of biological replicates, measurement repetitions, or time points for MPS studies. A dedicated MPS-focused group (CEN-CENELEC), founded in 2022 and involving members from Dynamic42 and esqLABS, is addressing this issue.

The PBPK models may be further improved by implementing more mechanistic detail on active drug transport, renal excretion and changes in metabolic functions during pregnancy. Transporter expression plays a crucial role in ADME processes by determining how drug compounds are absorbed across tissue barriers, distributed in the body, and eliminated. The use of the cell line Caco-2 as surrogate for intestinal epithelial cells is an adequate alternative used in a variety of *in vitro* models. However, important transporters such as MRP2-6, OATP-A/B, OCT1, and MCT1 were shown to be higher expressed compared to human small intestinal tissue, whereas BCRP levels were lower (Maubon et al., 2007). This discrepancy to human primary tissue can lead to deviations of how drugs are absorbed or released *in vivo*. Following studies should therefore include an in-depth characterization of transporter expression for a larger set of drug compounds and human primary tissue as cell source to enhance clinical translation. Additionally, studying donor-donor variability would elucidate the need to study subgroups of pregnant women or further individualize dosing regimen (Maass et al., 2015), (Kletting et al., 2015). Using isogenic OOC instead of models including non-donor-matched monocyte-derived macrophages (MDMs), could provide an even more complex, genetically tailored system and a better understanding of specific immune responses. However, for this study the scope was not to develop a new model but if existing, ready-to-use models can be interconnected and combined with computational modelling to show a benefit for preclinical testing. Although prednisone itself showed no stimulatory effect on isolated MDMs (Supplementary Figure S1B), it is known that the presence of MDMs in organ models affects enzyme expression and barrier integrity (Gröger et al., 2016), (Rennert et al., 2015). The absence of MDMs would likely have yielded different results, which might have been less reflective of *in vivo* conditions.

The integration of additional organ models would be worth considering for a more holistic ADME evaluation of prednisone. Organ models like heart, brain, or bone into the triple model could help to assess the systemic distribution of prednisone and to identify organ-specific toxicity or side effects. In addition, including the kidney may improve the predictive power of the model in terms of excretion, renal metabolism, and toxicity. However, adding more organ models increases system complexity, both in terms of engineering (creating functional models) and data interpretation. Understanding how the organs communicate (e.g., through the bloodstream or signaling molecules) is crucial. Handling this complexity must be carefully managed to ensure reliable and interpretable results.

The physiological relevance of the triple model used in this study is limited due to the use of cell lines and is a notable challenge for reproducibility and scalability of MPS. In example, BeWo cells might not fully replicate the phenotypic and functional characteristics of primary trophoblasts including differentiation, invasion, and hormone secretion. Another limitation is the exclusion of immune-hormonal interactions. The placenta is an immunologically active organ that must balance immune tolerance to the fetus while maintaining defense against pathogens. Our current model still does not to incorporate maternal immune cells and hormonal fluctuations, which are crucial for accurately modeling immune responses and endocrine signaling. However, our model offers a variety of future possibilities to cover these omissions making it reliable for studying conditions such as preeclampsia or infections that heavily involve immune-hormonal crosstalk effects on drug transfer. Our model introduces a dynamic system mimicking mechanical forces that influence placental physiology *in vivo* in comparison with current static setups that fail to mimic the circulatory exchange between maternal and fetal blood, which is critical for transport, elimination, and drug distribution. The absence of dynamic flow conditions may result in inaccurate representations of drug pharmacokinetics and toxicology, undermining the predictive value of these models for clinical applications. Nevertheless, our translation of MPS to humans has shown that transferability to the *in vivo* situation is possible and that highly complex and expensive models are not always necessary to obtain an initial assessment.

This overall outcome and potential is additionally highlighting the strength of our combined *in vitro–in silico* approach and of our biological model setup to potentially reduce animal testing in the framework of the 3Rs. Modelling the human placental interface preclinically is still highly challenging and comes with limitations*. In vitro* approaches highly focus on modelling the maternal–fetal interface (Cherubini et al., 2021), the placenta barrier itself, neglecting fundamental associated processes such as uptake, transport and distribution of a drug before reaching the placenta. Further, there are only a few animal models available to consider for preclinical testing, mostly of rodent or lagomorph origin (Carter, 2020). They can help to understand drug uptake and distribution, however, fail to recapitulate key features of the human placenta (Schmidt et al., 2015), (Schmidt et al., 2021). In regard to replacement efforts and the promotion of the use of new approach methods on regulatory level, our study falls into the scope of the FDA Modernization act 2.0 specifically supporting the use of organ-on-chip/microphysiological models and computer models, which we combined here, and FDA Modernization act 3.0 supporting the improvement in predictivity provided by non-clinical models, for which we gave evidence through the integration of computational modelling based on real experimental data. Our approach may also help to facilitate drug development and drug testing in regard to research addressing pregnant women. There are many scientific and ethical considerations, provided by regulators such as the FDA, to take care of. This involves preparing and providing all necessary patient information and documentation, providing justifications and including ethicists for study design, for example, rendering the whole process an administrative challenge and highly time consuming. A requirement for pre- and post-marketing clinical studies is as well to provide sufficient non-clinical data to justify the inclusion of pregnant women (Schmidt et al., 2015), (Schmidt et al., 2015), (Green et al., 2021). Here, our approach can provide a significant benefit to strengthen non-clinical data.

In modern drug development, computer-based models (“digital twins”) are increasingly used to enhance success rates, particularly through the integration and IVIVE (Przekwas et al., 2020), (Jones et al., 2015), (Murata et al., 2022) of *in vitro* data. This approach is gaining acceptance by regulatory bodies, including the FDA and EMA, for its ability to describe biological processes more accurately and make earlier, more precise predictions about drug efficacy and safety.

This approach is gaining momentum at the regulatory level. The European Union Reference Laboratory for Alternatives to Animal Testing (EURL ECVAM) is actively investigating the role of computational models in MPS and their acceptance by regulatory authorities. The recent passage of the FDA Modernization Act 2.0 further paves the way for the widespread adoption of MPS-computational approaches in drug development.

### 4.1 Conclusions

In conclusion, our integrated gut-liver-placenta model provides a powerful tool for investigating drug pharmacokinetics during pregnancy. By simulating the key processes of drug absorption, metabolism, and placental transfer, we demonstrated the system’s capability to predict fetal exposure to prednisone and its active metabolite prednisolone. Our results indicate the importance of selecting a context-of-use specific MPS, sampling schedule, and approach for translating to human pharmacology to accurately predict fetal compound exposures.

Looking ahead, the application of MPS technology—especially integrated multi-organ systems as demonstrated here—holds considerable potential for reducing the reliance on animal models in drug development. As more MPS are validated, their ability to accurately predict human outcomes will likely reduce the need for animal experimentation, addressing both ethical concerns and the high costs associated with traditional *in vivo* testing.

## Data Availability

The original contributions presented in the study are included in the article/Supplementary Material, further inquiries can be directed to the corresponding authors.
