# Supplementary material for: Digital twin-enhanced three-organ microphysiological system for studying drug pharmacokinetics in pregnant women
Source: Front Pharmacol. 2025 Feb 12;16:1528748. doi: 10.3389/fphar.2025.1528748 (PMC11873563; doi:10.3389/fphar.2025.1528748)
Supplement: Supplementary file 1 [file DataSheet2.docx]

**Supplementary Information S2 – Computational Modelling**

Containing Title: Digital Twin-Enhanced Three-Organ Microphysiological System for Studying Drug Pharmacokinetics in Pregnant Women

Authors: Katja Graf^1^, José Martin Murrieta-Coxca^2^, Tobias Vogt^1^, Sophie Besser^1^, Daria Geilen^1^, Tim Kaden^1,3^, Anne-Katrin Bothe^1^, Diana Maria Morales-Prieto^2^, Behnam Amiri^4,5^, Stephan Schaller^4^, Ligaya Kaufmann^6^, Martin Raasch^1*^, Ramy M Ammar^7,8#^, Christian Maass^4,5#*^

Affiliation

1 Dynamic42 GmbH, 07745 Jena, Germany

2 Placenta Lab, Department of Obstetrics, Jena University Hospital, 07747 Jena, Germany

3 Institute of Biochemistry II, Center for Sepsis Control and Care, Jena University Hospital, 07747 Jena, Germany

4 MPSlabs, ESQlabs GmbH, 26683 Saterland, Germany

5 ESQlabs GmbH, 26683 Saterland, Germany

6 Global Medical Affairs, Bayer Consumer Care AG, Basel, Switzerland

7 Global R&D, Bayer Consumer Health, Steigerwald Arzneimittelwerk GmbH, Havelstraße 5, 64295 Darmstadt, Germany

8 Department of Pharmacology and Toxicology, Faculty of Pharmacy, Kafrelsheikh University, Kafr-El Sheikh 33516, Egypt

* corresponding author

# co-shared last authorship

Keywords: pregnancy, organ-on-chip, computational modelling, pharmacokinetics, safety

## Predicting Prednisone PK in Pregnant Women

##
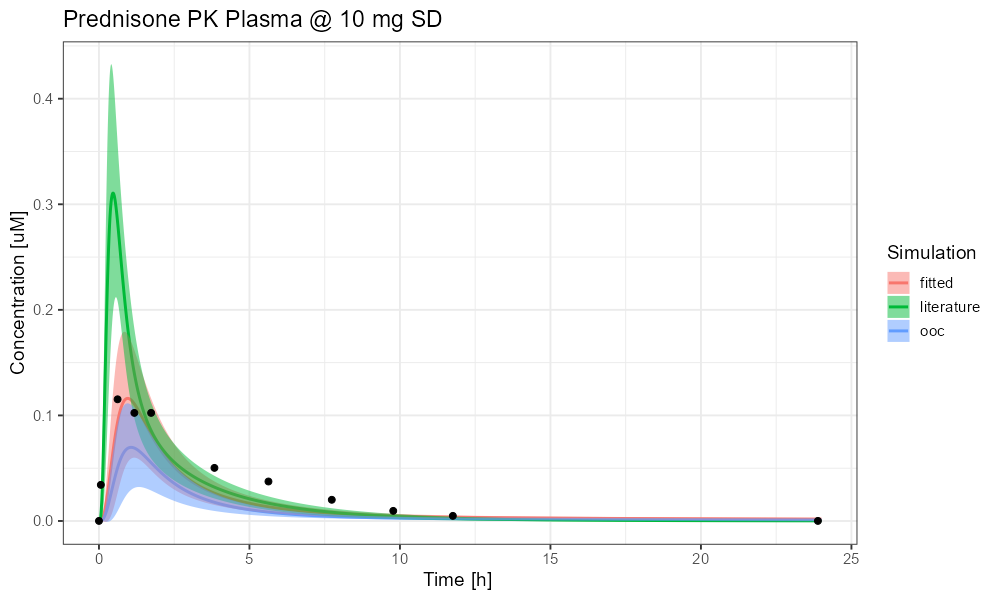


**Supplementary Fig. 2.1:** **A PBPK model was used to simulate the kinetics of prednisone in n = 100 pregnant women.**Model simulations (lines) were compared to clinical data (black dots) using either fitted clearance and absorption rates (red curve), literature-based values (green curve) or the MPS-derived values (blue curve). Overall, the fitted curves best match the observed data, while the MPS-derived values come close to this description. Interestingly, reported literature values did overpredict the concentration maximum of prednisone quite considerably. Shaded areas represent +/- 1 standard deviation.


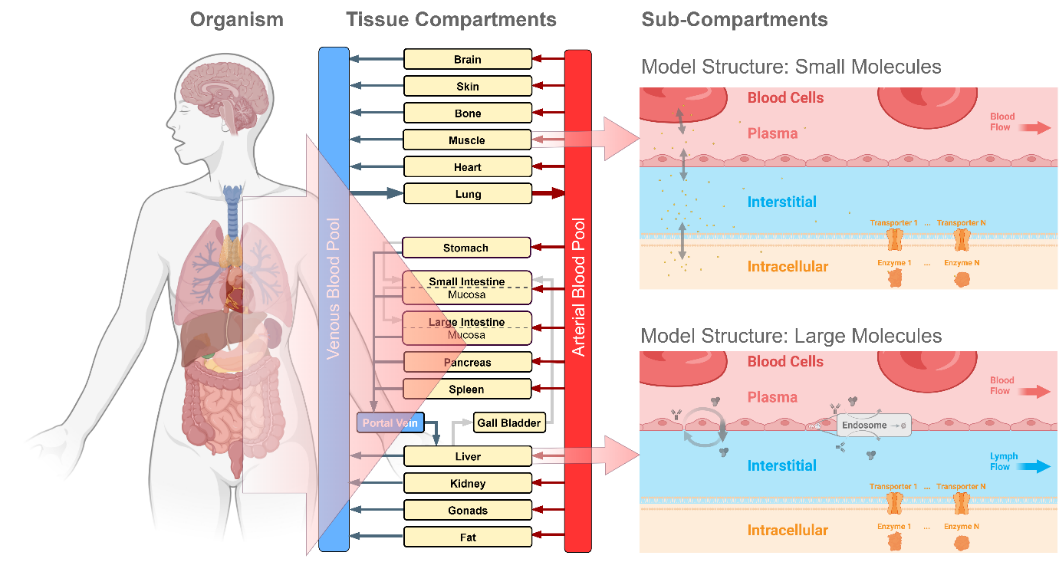


**Supplementary Fig. 2.2:** **Generic overview of the PBPK model approach.**
Human physiology is represented as compartments (colored boxes) and connected via blood flow. The model uses ordinary differential equations to describe mass-balance between compartments. Further, every organ comprises sub-compartments representing intracellular and interstitial spaces. Flow rates, permeability, and partitioning between these compartments are informed by physico-chemical properties of the investigated compounds.

## Digital twinning approach

The **digital twinning approach** in this context refers to creating a virtual model (or “twin”) of a biological system that mirrors its physical counterpart. Specifically, in pharmacokinetic (PK) studies, it simulates the absorption, distribution, metabolism, and excretion (ADME) of drugs in humans. The key elements of this approach are:

1. **Integration of experimental and in silico data**: The digital twin leverages data from in vitro systems, such as microphysiological systems (MPS), organ-on-chip models, or traditional cell cultures. In the context of this study, data from a three-organ MPS (gut, liver, and placenta) is used to inform the model.
2. **Mathematical modeling**: The digital twin applies physiologically based pharmacokinetic (PBPK) models to simulate drug behavior. The PBPK model represents the human body as compartments corresponding to organs/tissues (e.g., gut, liver, placenta) and captures how drugs are absorbed, metabolized, and excreted over time. Each compartment is governed by differential equations that describe drug movement between tissues and organs.
3. **Parameter Fitting**: Experimental data from MPS informs key parameters (e.g., gut permeability, liver clearance) in the PBPK model. This step allows the model to reflect specific biological processes under study. The parameter fitting ensures that the digital twin can closely match experimental and clinical data (e.g., plasma concentrations).

**Key Equations Used in the Digital Twin Approach**

**Compartmental Model**

Each organ (gut, liver, placenta) in the PBPK model is treated as a compartment. The concentration of a drug in a compartment, CiC_iCi​, is modeled using differential equations that describe the change in drug concentration over time due to various processes (absorption, metabolism, and transfer between compartments).

- **Mass balance equation** for a compartment iii:

# Differential equation for drug concentration in a given compartment i

dC_i_dt <- (Q_i / V_i) * (C_in - C_i) - Clearance_i * C_i

Where:

- Q_i is the blood flow rate into the compartment
- V_i is the volume of the compartment
- C_in is the drug concentration entering the compartment
- C_i is the drug concentration in the compartment
- Clearance_i is the clearance rate in compartment

**Liver Digital Twin with Prednisone Metabolism to Prednisolone**

The liver compartment is one of the most important in the digital twin, as it handles both the metabolism of prednisone into prednisolone and the excretion of the active drug. The differential equations govern the drug concentrations in various compartments within the liver, accounting for interstitial, cellular, and plasma compartments.

- **dc1**: Governs the mixing of prednisone between the plasma reservoir and the liver's interstitial space.
- **dc2**: Models the movement of prednisone between the liver's interstitial space and the top channel, which contains liver sinusoidal endothelial cells (LSECs) and macrophages (MPh). This equation also models drug exchange between the interstitial space and the cellular compartment.
- **dc3**: Describes the transport of prednisone between the liver interstitium and liver cells through permeability pathways (via intercellular and transcellular routes). This equation also accounts for the partitioning between the plasma and cellular spaces.
- **dc4**: Describes the concentration of prednisone within liver cells and its metabolism. The free intracellular fraction (fucellfu_{cell}fucell​) undergoes clearance through metabolism. Prednisolone is generated during this step.
- **dc5**: Describes the movement of prednisone from the liver interstitium into the bottom channel containing hepatocytes (Heps), reflecting drug clearance and flow within the liver.

For **prednisolone**, generated during prednisone metabolism:

- **dc6**: Governs the mixing of prednisolone in the plasma reservoir.
- **dc7**: Describes the movement of prednisolone between the plasma and the top channel (LSECs + MPh).
- **dc8**: Describes prednisolone transport between the liver's top and bottom channels, and the intracellular metabolism, with clearance rates specific to prednisolone.

#############

dc1 <- Q_mix*(c2 - c1)/V_mix # mixer, reservoir

dc2 <- Q_mix*(c1 - c2)/V_top + P*SA*(c3/K_int_pls - c2)/V_top # top channel (LSECs + MPh)

dc3 <- (P*SA*(c5 - c3 / K_int_pls) + P*SA*(c2 - c3 / K_int_pls) - K_water_int * PA_int_cell * c3 + K_water_cell * PA_cell_int * c4)/V_interstitium

dc4 <- fu_cell*(K_water_int * PA_int_cell * c3 - K_water_cell * PA_cell_int * c4)/V_liver_cell - c4 * Cl/V_liver_cell

dc5 <- (P*SA*(c3 / K_int_pls - c5 ))/V_bottom # bottom channel (Heps)

# prednisolone

dc6 <- Q_mix*(c7 - c6)/V_mix

dc7 <- Q_mix*(c6 - c7)/V_top + P_pl*SA*(c8 - c7)/V_top

dc8 <- P_pl*SA*(c7 - c8)/V_bottom + Cl*c4/V_bottom - Cl_pred*c8/V_bottom

**Gut Digital Twin**

The gut compartment is responsible for the **absorption** of prednisone into the body following oral administration. The drug passes through the intestinal wall and moves between different compartments (epithelial cells, interstitium, and vasculature).

- **dc1**: Models the mixing of prednisone between the plasma reservoir and the top channel.
- **dc2**: Describes drug movement between the top channel (intestinal epithelial cells, HUVECs, macrophages) and the vascular compartment.
- **dc3**: Describes the transfer between the interstitium and other compartments through permeability pathways and water exchange processes.
- **dc4**: Represents intracellular drug concentration and clearance within gut cells.
- **dc5**: Models the mixing between the plasma reservoir and bottom channel, describing the distribution across the entire gut layer.

############

dc1 <- Q_mix_top*(c2 - c1)/V_mix_top # mixer, top reservoir

dc2 <- Q_mix_top*(c1 - c2)/V_top + SA*(P_EV*c3 / K_int_pls - P_VE*c2)/V_top # top channel (HUVECs + MPh)

dc3 <- (SA*(P_VE*c2 - P_EV*c3 / K_int_pls) - K_water_int * PA_int_cell * c3 + K_water_cell * PA_cell_int * c4 + SA*(P_VE*c5 - P_EV*c3 / K_int_pls))/V_interstitium

dc4 <- (K_water_int * PA_int_cell * c3 - K_water_cell * PA_cell_int * c4)/V_cell - c4 * Cl/V_cell

dc5 <- Q_mix_bottom*(c6 - c5)/V_bottom + SA*(P_EV*c3 / K_int_pls - P_VE*c5)/V_bottom # bottom channel (HUVECs + MPh)

dc6 <- Q_mix_bottom*(c5 - c6)/V_mix_bottom

**Placenta Digital Twin**

The placenta, like the liver, is modeled as a barrier where the drug crosses from the maternal to the fetal compartments. However, unlike the liver, the placenta does not metabolize prednisone or generate prednisolone. The equations reflect the movement of the drug across the placental barrier without metabolic processes.

- The equations for the placenta are similar to the liver model but without terms for metabolic clearance or prednisolone generation. This means only permeability and partitioning processes govern the transfer of prednisone.

**Table 1: Overview of model parameters used to develop the chip-specific digital twins.**

|  | Parameter | Unit | Gut | Liver | Placenta |
| --- | --- | --- | --- | --- | --- |
| Top channel | V_top | ml | 0.14 | 0.14 | 0.12 |
| Bottom channel | V_bottom | ml | 0.07 | 0.15 | 0.07 |
| Reservoir | V_mix | ml | 3.0 | 0.8 | 0.6 |
| Surface area | SA | cm2 | 1.32 | 1.32 | 1.02 |
| Flow rate | Qmix | ml/min | 0.025 | 0.025 | 0.025 |

**Summary of the Digital Twin Approach**

In the **Digital Twin** framework, each compartment (gut, liver, and placenta) is represented by ordinary differential equations (ODEs) that simulate how drug concentrations change over time. The compartments are linked together through blood flow (mixing terms Q) and permeability parameters that describe drug movement between compartments. By adjusting parameters (e.g., gut permeability, liver clearance), the digital twin is fitted to experimental and clinical data, allowing predictions of maternal and fetal drug concentrations over time. The final goal is to simulate drug pharmacokinetics and optimize treatment strategies for pregnant women using this in silico approach.

**Digital Twin Parameters**

Parameters used in the digital twin approach are informed by physico-chemical properties, which are used to estimate downstream parameters in PK-Sim® as follows:

- 1. Liver-Chip /Placenta-Chip

K_int_pls = getParameter("Neighborhoods|Periportal_pls_Periportal_int|Drug|Partition coefficient (interstitial/plasma)", sim)

P_endothelial = getParameter("Neighborhoods|Periportal_pls_Periportal_int|Drug|P (plasma<->interstitial)",sim)

SA = getParameter("Neighborhoods|Periportal_pls_Periportal_int|Surface area (plasma/interstitial)",sim)

K_water_int = getParameter("Neighborhoods|Periportal_int_Periportal_cell|Drug|Partition coefficient (interstitial/water)", sim)

K_water_cell = getParameter("Neighborhoods|Periportal_int_Periportal_cell|Drug|Partition coefficient (intracellular/water)", sim)

PA_cell_int = getParameter("Neighborhoods|Periportal_int_Periportal_cell|Drug|P*SA intracellular -> interstitial",sim)

PA_int_cell = getParameter("Neighborhoods|Periportal_int_Periportal_cell|Drug|P*SA interstitial -> intracellular",sim)

- 1. Gut-Chip

K_int_pls = getParameter("Neighborhoods|SmallIntestine_pls_SmallIntestine_int|Drug|Partition coefficient (interstitial/plasma)", sim)

P_endothelial = getParameter("Neighborhoods|SmallIntestine_pls_SmallIntestine_int|Drug|P (plasma<->interstitial)",sim)

SA = getParameter("Neighborhoods|SmallIntestine_pls_SmallIntestine_int|Surface area (plasma/interstitial)",sim)

K_water_int = getParameter("Neighborhoods|SmallIntestine_int_SmallIntestine_cell|Drug|Partition coefficient (interstitial/water)", sim)

K_water_cell = getParameter("Neighborhoods|SmallIntestine_int_SmallIntestine_cell|Drug|Partition coefficient (intracellular/water)", sim)

PA_cell_int = getParameter("Neighborhoods|SmallIntestine_int_SmallIntestine_cell|Drug|P*SA intracellular -> interstitial",sim)

PA_int_cell = getParameter("Neighborhoods|SmallIntestine_int_SmallIntestine_cell|Drug|P*SA interstitial -> intracellular",sim)

Where K refers to the partitioning coefficient, P to permeability, PA to permeability surface area, and SA to surface area.

## R Code to run PBPK model simulations

Following is the R code used to simulate the kinetics of both prednisone and prednisolone in pregnant women:

###########################################################

# Loading an existing simulation file

sim = loadSimulation("Pregnant Women.pkml")

idx = 1

method <- c('ooc')

# Setting key parameter values for the simulation

placenta_pc_value <- c(1.5) # Partition coefficient for the placenta

gut_perm_value <- c(2.9)*1E-7 # Gut permeability cm/min

liver_met_value <- c(12.4)/1000 # Liver clearance rate ml/min/kg

# Updating parameters in the simulation model with new values

placenta_pc <- getParameter("Neighborhoods|PlacentaMaternal_pls_PlacentaFetal_cell|Prednisone|Partition coefficient (intracellular/plasma)", sim)

newValue <- toBaseUnit(quantity = placenta_pc, values = placenta_pc_value[idx], unit = "")

setParameterValues(parameters = placenta_pc, values = newValue)

placenta_pc <- getParameter("Neighborhoods|PlacentaMaternal_pls_PlacentaFetal_cell|Prednisolone|Partition coefficient (intracellular/plasma)", sim)

newValue <- toBaseUnit(quantity = placenta_pc, values = placenta_pc_value[idx], unit = "")

setParameterValues(parameters = placenta_pc, values = newValue)

gut_perm <- getParameter("Prednisone|Specific intestinal permeability (transcellular)", sim)

newValue <- toBaseUnit(quantity = gut_perm, values = gut_perm_value[idx], unit = "dm/min")

setParameterValues(parameters = gut_perm, values = newValue)

liver_met <- getParameter("Prednisone-Total Hepatic Clearance-fitted|Plasma clearance", sim)

newValue <- toBaseUnit(quantity = liver_met, values = liver_met_value[idx], unit = "l/min/kg")

setParameterValues(parameters = liver_met, values = newValue)

# Setting simulation time to 100 days

sim_time = 100 # days

setOutputInterval(simulation = sim, startTime = 0, endTime = sim_time*24*60, resolution = 2) # in minutes

# Running the simulation with the population

simulationResults_Pop_real <- runSimulation(simulation = sim, population = myPopulation$population)

list_real = simulationResults_Pop_real$allQuantityPaths

ids = 0:(numberOfIndividuals-1)

final_fetal_plasma = list()

final_fetal_tissue = list()

final_maternal_plasma = list()

final_fetal_prednisolone_plasma = list()

final_maternal_prednisolone_plasma = list()

for (idxx in ids){

# Extracting plasma and tissue concentration data from the simulation results

resultsPath_Pop <- simulationResults_Pop_real$allQuantityPaths[17]

resultsData_Pop <- getOutputValues(simulationResults_Pop_real, quantitiesOrPaths = resultsPath_Pop)

final_fetal_plasma[idxx+1] <- list(resultsData_Pop$data$"Organism|Fetus|Plasma|Prednisone|Concentration in container"[resultsData_Pop$data$IndividualId==idxx])

resultsPath_Pop <- simulationResults_Pop_real$allQuantityPaths[23]

resultsData_Pop <- getOutputValues(simulationResults_Pop_real, quantitiesOrPaths = resultsPath_Pop)

final_fetal_tissue[idxx+1] <- list(resultsData_Pop$data$"Organism|Fetus|Prednisone|Tissue" [resultsData_Pop$data$IndividualId==idxx])

resultsPath_Pop <- simulationResults_Pop_real$allQuantityPaths[1]

resultsData_Pop <- getOutputValues(simulationResults_Pop_real, quantitiesOrPaths = resultsPath_Pop)

final_maternal_plasma[idxx+1] <- list(resultsData_Pop$data$"Organism|PeripheralVenousBlood|Prednisone|Plasma (Peripheral Venous Blood)"[resultsData_Pop$data$IndividualId==idxx])

resultsPath_Pop <- simulationResults_Pop_real$allQuantityPaths[19]

resultsData_Pop <- getOutputValues(simulationResults_Pop_real, quantitiesOrPaths = resultsPath_Pop)

final_fetal_prednisolone_plasma[idxx+1] <- list(resultsData_Pop$data$"Organism|Fetus|Plasma|Prednisolone|Concentration in container"[resultsData_Pop$data$IndividualId==idxx])

resultsPath_Pop <- simulationResults_Pop_real$allQuantityPaths[2]

resultsData_Pop <- getOutputValues(simulationResults_Pop_real, quantitiesOrPaths = resultsPath_Pop)

final_maternal_prednisolone_plasma[idxx+1] <- list(resultsData_Pop$data$"Organism|PeripheralVenousBlood|Prednisolone|Plasma (Peripheral Venous Blood)"[resultsData_Pop$data$IndividualId==idxx])

print(idxx) # For tracking progress

}

single_time = resultsData_Pop$data$Time[resultsData_Pop$data$IndividualId==idxx] # Retrieve time vector

# Convert results to data frames and compute means and standard deviations

final_fetal_plasma = data.frame(final_fetal_plasma)

final_fetal_plasma_mean = rowMeans(final_fetal_plasma)

final_fetal_plasma_std = rowSds(as.matrix(final_fetal_plasma), na.rm=TRUE)

final_fetal_tissue = data.frame(final_fetal_tissue)

final_fetal_tissue_mean = rowMeans(final_fetal_tissue)

final_fetal_tissue_std = rowSds(as.matrix(final_fetal_tissue), na.rm = TRUE)

final_maternal_plasma = data.frame(final_maternal_plasma)

final_maternal_plasma_mean = rowMeans(final_maternal_plasma)

final_maternal_plasma_std = rowSds(as.matrix(final_maternal_plasma), na.rm = TRUE)

final_fetal_prednisolone_plasma = data.frame(final_fetal_prednisolone_plasma)

final_fetal_prednisolone_plasma_mean = rowMeans(final_fetal_prednisolone_plasma)

final_fetal_prednisolone_plasma_std = rowSds(as.matrix(final_fetal_prednisolone_plasma), na.rm = TRUE)

final_maternal_prednisolone_plasma = data.frame(final_maternal_prednisolone_plasma)

final_maternal_prednisolone_plasma_mean = rowMeans(final_maternal_prednisolone_plasma)

final_maternal_prednisolone_plasma_std = rowSds(as.matrix(final_maternal_prednisolone_plasma), na.rm = TRUE)

#---------------------###############---------------------#

# plotting

#---------------------###############---------------------#

# prednisone

colors = c("Fetal" = "blue","Maternal" = "red")

pp <- ggplot(df) +

geom_line(aes(x=(single_time/(60*24)), y=`Fetal Plasma`,color = "Fetal"), df,size = 2)+

geom_ribbon(aes(x=(single_time/(60*24)),ymin = (df$`Fetal Plasma` - df$`FP Std`),ymax = (df$`Fetal Plasma` + df$`FP Std`)),fill = 'lightsteelblue',alpha = 0.5)+

geom_line(aes(x=(single_time/(60*24)), y=`Maternal Plasma`,color = "Maternal"), df,size = 1,alpha = 0.5) +

geom_ribbon(aes(x=(single_time/(60*24)),ymin = (df$`Maternal Plasma` - df$`MP Std`),ymax = (df$`Maternal Plasma` + df$`MP Std`)),fill = 'coral',alpha = 0.5) +

labs(x = "Time [day] ", y = 'Concentration [uM]', title = 'Prednisone PK Plasma @ 10 mg SD',color = "Legend") + scale_color_manual(values = colors) +

theme_bw(base_size = 24) +

ylim(0,0.5)+

theme(

legend.position = "right",

legend.justification = "left",

legend.direction = "vertical"

)

pp

#figure_file = paste0(method[idx],'fetal_maternal_prednisone.tiff')

#ggsave(file= figure_file, plot=pp, width = 10, height = 6, dpi = 300, units = "in")

# prednisolone

colors = c("Fetal Plasma" = "green","Maternal Plasma" = "red")

pg <- ggplot(df) +

geom_line(aes(x=(single_time/(60*24)), y=`Fetal Plasma Prednisolone`,color = "Fetal Plasma"), df,size = 2)+

geom_ribbon(aes(x=(single_time/(60*24)),ymin = (df$`Fetal Plasma Prednisolone` - df$`FPP Std`),ymax = (df$`Fetal Plasma Prednisolone` + df$`FPP Std`)),fill = 'lightgreen',alpha = 0.5)+

geom_line(aes(x=(single_time/(60*24)), y=`Maternal Plasma Prednisolone`,color = "Maternal Plasma"), df,size = 1,alpha = 0.5) +

geom_ribbon(aes(x=(single_time/(60*24)),ymin = (df$`Maternal Plasma Prednisolone` - df$`MPP Std`),ymax = (df$`Maternal Plasma Prednisolone` + df$`MPP Std`)),fill = 'coral',alpha = 0.5) +

labs(x = "Time [day] ", y = 'Concentration [uM]', title = 'Prednisolone PK Plasma @ 10 mg SD',color = "Legend") + scale_color_manual(values = colors) +

theme_bw(base_size = 24) +

ylim(0,0.5)+

theme(

legend.position = "right",

legend.justification = "left",

legend.direction = "vertical"

)

pg

#figure_file = paste0(method[idx],'fetal_maternal_prednisolone.tiff')

#ggsave(file= figure_file, plot=pg, width = 10, height = 6, dpi = 300, units = "in")

colors = c("Prednisolone" = "blue","Prednisone" = "green")

pig <- ggplot(df) +

geom_line(aes(x=(single_time/(60*24)), y=`Fetal Plasma Prednisolone`,color = "Prednisolone"), df,size = 2)+

geom_ribbon(aes(x=(single_time/(60*24)),ymin = (df$`Fetal Plasma Prednisolone` - df$`FPP Std`),ymax = (df$`Fetal Plasma Prednisolone` + df$`FPP Std`)),fill = 'lightsteelblue',alpha = 0.5)+

geom_line(aes(x=(single_time/(60*24)), y=`Fetal Plasma`,color = "Prednisone"), df,size = 2)+

geom_ribbon(aes(x=(single_time/(60*24)),ymin = (df$`Fetal Plasma` - df$`FP Std`),ymax = (df$`Fetal Plasma` + df$`FP Std`)),fill = 'lightgreen',alpha = 0.5)+

labs(x = "Time [day] ", y = 'Concentration [uM]', title = 'Fetal Plasma PK @ 10 mg SD',color = "Legend") + scale_color_manual(values = colors) +

theme_bw(base_size = 24) +

ylim(0,0.2)+

theme(

legend.position = "right",

legend.justification = "left",

legend.direction = "vertical"

)

pig

#figure_file = paste0(method[idx],'Fetal_comparison.tiff')

#ggsave(file= figure_file, plot=pig, width = 10, height = 6, dpi = 300, units = "in")

#---------------------###############---------------------#

Cmax_fetal_maternal = (max(df$`Fetal Plasma`)/max(df$`Maternal Plasma`))

Cmax_fetal_maternal

Cmax_fetal_maternal_prednisolone = (max(df$`Fetal Plasma Prednisolone`)/max(df$`Maternal Plasma Prednisolone`))

Cmax_fetal_maternal_prednisolone
