# Supplementary material for: Digital twin-enhanced three-organ microphysiological system for studying drug pharmacokinetics in pregnant women
Source: Front Pharmacol. 2025 Feb 12;16:1528748. doi: 10.3389/fphar.2025.1528748 (PMC11873563; doi:10.3389/fphar.2025.1528748)
Supplement: Supplementary file 2 [file DataSheet1.docx]

**Supplementary Information S1 – Biology**

Containing Title: Digital Twin-Enhanced Three-Organ Microphysiological System for Studying Drug Pharmacokinetics in Pregnant Women

Authors: Katja Graf^1^, José Martin Murrieta-Coxca^2^, Tobias Vogt^1^, Sophie Besser^1^, Daria Geilen^1^, Tim Kaden^1,3^, Anne-Katrin Bothe^1^, Diana Maria Morales-Prieto^2^, Behnam Amiri^4,5^, Stephan Schaller^4^, Ligaya Kaufmann^6^, Martin Raasch^1*^, Ramy M Ammar^7,8#^, Christian Maass^4,5#*^

Affiliation

1 Dynamic42 GmbH, 07745 Jena, Germany

2 Placenta Lab, Department of Obstetrics, Jena University Hospital, 07747 Jena, Germany

3 Institute of Biochemistry II, Center for Sepsis Control and Care, Jena University Hospital, 07747 Jena, Germany

4 MPSlabs, ESQlabs GmbH, 26683 Saterland, Germany

5 ESQlabs GmbH, 26683 Saterland, Germany

6 Global Medical Affairs, Bayer Consumer Care AG, Basel, Switzerland

7 Global R&D, Bayer Consumer Health, Steigerwald Arzneimittelwerk GmbH, Havelstraße 5, 64295 Darmstadt, Germany

8 Department of Pharmacology and Toxicology, Faculty of Pharmacy, Kafrelsheikh University, Kafr-El Sheikh 33516, Egypt

* corresponding author

# co-shared last authorship

Keywords: pregnancy, organ-on-chip, computational modelling, pharmacokinetics, safety


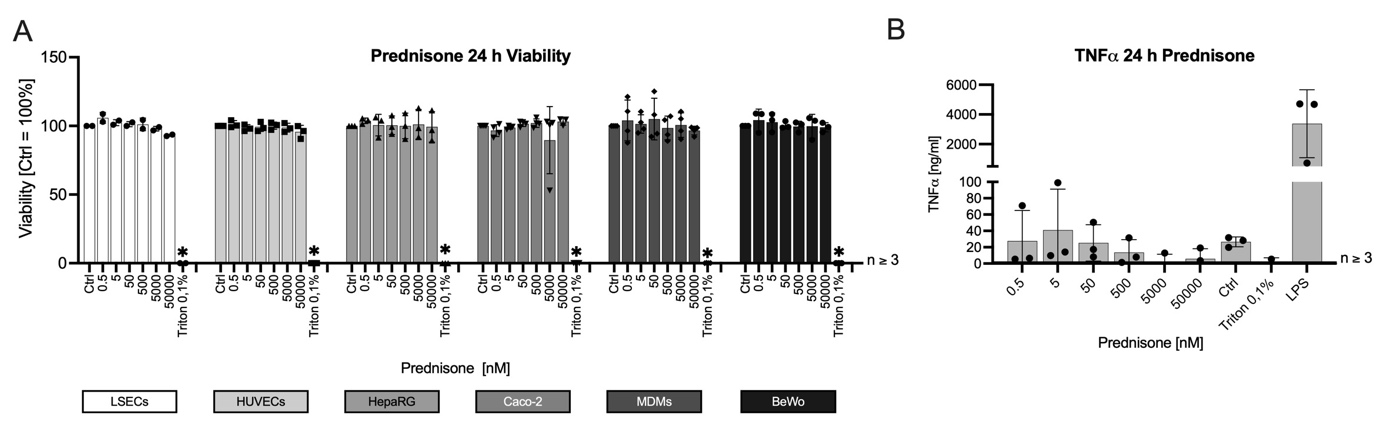


**Supplementary Fig. 1: Treatment with prednisone (PD) does not impair the viability of cells cultured in 2D, nor does it lead to activation of immune cells.**
**A**: To determine the effects of PD on the viability of the individual cell types used in the organ models, the cells were treated with PD containing medium (0.5 - 50000 nM) for 24 h. As a positive control for cell damage, the cells were treated for 10 min with medium containing 0.1 % Triton-X-100. Compared to the untreated control (Ctrl, medium containing only solvent (DMSO= 0.1%)), no significant change in viability was measured by PD treatment. Data shown are mean +/− S.D; n ≥ 3; one-way ANOVA, all conditions DF = 7; LSECs: F = 561.9, HUVECs: F = 907.4, HepaRG: F = 161.9, Caco-2: F = 1199, MDMs: F = 119.1, BeWos: F = 286.2, p (*) < 0.001. **B**: TNFα levels were measured in culture supernatant of MDMs treated with control (Ctrl, 0.1% DMSO), PD (0.5 - 50000 nM), LPS (100 ng/mL) for 24 h or 0.1% Triton-X-100 for 10 min via ELISA. Only LPS as a positive control of immune cell activation led to pronounced TNFα release. TNFα levels after PD treatment were comparable to untreated controls. Data shown are mean +/− S.D; n ≥ 3; one-way ANOVA, DF = 9; F = 6.48, p < 0.05.

**Supplementary Materials and Methods**

**Viability assay**

Cells were seeded in collagen A (50 µg/mL)-coated 96-well microplates (Caco-2/HepaRG/LSECs/HUVECs/BeWos: 3 × 10^4^ cells/well, MDMs: 4 × 10^4^ cells/well) in 200 µL of GPM, HPM, ECGM MV, and VPM, respectively. After 24 h of pre-culture medium was exchanged and cells were treated with different prednisone concentrations in cell-specific medium (0.5 nM to 50 000 nM) for 24 h. As a positive control for cell damage, the cells were treated for 10 min before analysis with a medium containing 0.1 % Triton-X-100.

Viability was measured by using the CellTiter-Glo Luminescent Cell Viability Assay (Promega, Walldorf, Germany). The assay was performed following the protocol provided by the manufacturer. Briefly, medium was replaced by phenol red-free William’s Medium E (PAN-Biotech) and microplates were equilibrated for 30 min at RT. CellTiter-Glo Reagent was added at a ratio of 1:1 to the wells. Cells were incubated on an orbital shaker for 2 min and 10 min without shaking in the dark to stabilize the luminescence signal. Luminescence was measured with an integration time of 1000 ms using a microplate reader (INFINITE 200 PRO, Tecan, Crailsheim, Germany).

**Cytokine analysis**

MDMs were seeded in collagen A (50 µg/mL)-coated 96-well microplates at 4 × 10^4^ cells/well in 200 µL of VPM. After 24 h of pre-culture medium was exchanged and cells were treated with different prednisone concentrations in VPM (0.5 nM to 50 000 nM) for 24 h. As a positive controls, the cells were treated either for 10 min before analysis with a medium containing 0.1 % Triton-X-100 (cell damage) or for 24 h with medium containing 100 ng/mL LPS (Merck, Darmstadt, Germany / immune cell activation).

TNFα release was quantified in cell culture supernatants using ELISA MAX™ Deluxe Set Human TNFα (BioLegend, San Diego, CA, USA). The assay was performed according to the manufacturer’s instructions. Absorption was measured at 450 nm with a reference wavelength of 650 nm in a microplate reader (INFINITE 200 PRO, Tecan). TNFα concentrations were calculated from a TNFα standard curve.

**Supplementary Table 1: List of antibodies and used concentrations**

| **Compart-ment** | **Primary Antibody** | **Type/ reactivity** | **Final Concentra-tion** | **Manufacturer** | **Article number** |
| --- | --- | --- | --- | --- | --- |
| Vascular  (HUVECs /LSECs + MDMs) | Anti-Mannose Receptor (CD206) | rabbit/ human | 0.9 µg/mL | Abcam | Ab209327 |
|  | Anti-CD31 | mouse/ human | 0.5 µg/mL | Cell Signaling Technology (Leiden, The Netherlands) | 3528 |
|  | Anti-VE-Cadherin | goat/ human | 2 µg/mL | Bio-Techne (Minneapolis, USA) | AF938 |
| Epithelial  (Caco-2) | Anti-E-Cadherin | mouse/ human, mouse, rat | 2.5 µg/mL | BD –Bioscience (Heidelberg, Germany) | 610182 |
|  | Anti-ZO-1 | rabbit/ human | 1.25 µg/mL | Thermo Fisher Scientific | 61-7300 |
| Epithelial  (HepaRG) | Anti-Cytochrome P450 Enzyme (CYP3A4) | rabbit/ human, pig | 10-15 µg/mL | Merck | AB1254 |
|  | Anti-Glutathione Transferase Alpha (a-GST) | goat/ human, rat | 136 µg/mL | Biozol (Eching, Germany) | GS09 |
|  | Anti-Asialogycopro-tein receptor 1 (ASGPR1) | mouse/ human, rat | 2 µg/mL | BD Bioscience | 563654 |
| Maternal  (BeWos) | Anti-b-Catenin (Alexa Fluor 647 Conjugated) | mouse/human/dog/ rat | 0.5 µg/mL | Cell Signaling | 4627 |
|  | Anti-ZO-1 (Alexa Fluor 488 Conjugated) | mouse/human/dog/ rat | 0.5 µg/mL | Thermo Fisher Scientific | 339188 |
| Fetal  (HUVECs) | Anti-CD31 (Alexa Fluor 488 Conjugated) | mouse/ human | 0.5 µg/mL | Thermo Fisher Scientific | MA-18135 |
|  | Anti-vWF (Alexa Fluor 647 Conjugated) | rabbit/human | 0.5 µg/mL | Abcam | ab195029 |
| Secondary Antibody | Donkey anti-Mouse IgG (H+L), Alexa Flour 555 | | Thermo Fisher Scientific | | A-31570 |
|  | Donkey anti-Rabbit IgG (H+L), Alexa Flour 488 | | Thermo Fisher Scientific | | A-32790 |
|  | Donkey anti-Goat IgG (H+L), Alexa Flour 647 | | Thermo Fisher Scientific | | A-21447 |
|  | 4’,6-diamidino-2-phenylindole (DAPI) | | Thermo Fisher Scientific | | D1306 |

***Supplementary Table 2: Prednisone and Prednisolone concentrations in cell culture supernatants collected after 10 h, 20 h, and 24 h of treatment determined by LC/MS***
